# Human aromatic amino acid decarboxylase is an asymmetric and flexible enzyme: Implication in aromatic amino acid decarboxylase deficiency

**DOI:** 10.1002/pro.4732

**Published:** 2023-08-01

**Authors:** Giovanni Bisello, Rui P. Ribeiro, Massimiliano Perduca, Benny Danilo Belviso, Patrizia Polverino de' Laureto, Alejandro Giorgetti, Rocco Caliandro, Mariarita Bertoldi

**Affiliations:** ^1^ Department of Neuroscience, Biomedicine and Movement Sciences Section of Biological Chemistry Verona Italy; ^2^ Department of Biotechnology University of Verona Verona Italy; ^3^ National Research Council (CNR) Institute of Crystallography, CNR Bari Italy; ^4^ Department of Pharmaceutical and Pharmacological Sciences University of Padova Padova Italy

**Keywords:** AADC deficiency, human aromatic amino acid decarboxylase, L‐Dopa methylester, molecular dynamics, small angle x‐ray scattering, x‐ray diffraction

## Abstract

Human aromatic amino acid decarboxylase (AADC) is a pyridoxal 5′‐phosphate‐dependent enzyme responsible for the biosynthesis of dopamine and serotonin, essential neurotransmitters involved in motor and cognitive abilities. Mutations in its gene lead to AADC deficiency, a monogenic rare neurometabolic childhood parkinsonism characterized by severe motor and neurodevelopmental symptoms. Here, for the first time, we solved the crystal structure of human holoAADC in the internal aldimine (1.9 Å) and in the external aldimine (2.4 Å) of the substrate analog L‐Dopa methylester. In this intermediate, the highly flexible AADC catalytic loop (CL) is captured in a closed state contacting all protein domains. In addition, each active site, composed by residues of both subunits, is connected to the other through weak interactions and a central cavity. By combining crystallographic analyses with all‐atom and coarse‐grained molecular dynamics simulations, SAXS investigations and limited proteolysis experiments, we realized that the functionally obligate homodimeric AADC enzyme in solution is an elongated, asymmetric molecule, where the fluctuations of the CL are coupled to flexibility at the edge between the N‐terminal and C‐terminal domains. The structural integrity of this peripheral protein region is essential to catalysis, as assessed by both artificial and 37 AADC deficiency pathogenic variants leading to the interpretation that structural dynamics in protein regions far from the active site is essential for CL flexibility and the acquirement of a correct catalytically competent structure. This could represent the molecular basis for pathogenicity prediction in AADC deficiency.

## 1 INTRODUCTION

Dopamine and serotonin play a crucial role as neurotransmitters in human motor function and neurocognitive development (Ng et al., 2015). They are produced by decarboxylation of the related amino acids, L‐3′,4′‐dihydroxyphenylalanine (L‐Dopa) and L‐5′‐hydroxytryptophan (L‐5HTP), catalyzed by the pyridoxal 5′‐phosphate (PLP)‐dependent enzyme aromatic amino acid decarboxylase (AADC; E.C. 4.1.1.28). AADC plays a central role in monoaminergic neurometabolic synthesis and depletion of monoamine neurotransmitters such as dopamine leads to severe disorders. These include Parkinson's disease (PD), due to the progressive loss of brain dopaminergic neurons, and AADC deficiency (AADCd) caused by mutations in the *DDC* gene coding for AADC. Although deeply different and characterized by a plethora of broad‐range symptoms, these pathological conditions share a variety of clinical signs including severe hypotonia, bradykinesia and neurodevelopmental disorders. However, differently from neurodegenerative PD that mainly affects adults, AADCd is an inherited rare neurotransmitter disease (Himmelreich et al., 2019) manifesting in childhood (Leuzzi et al., 2021).

One of the approaches to tackle AADC deficiency is to understand the molecular basis for the altered behavior of the enzyme by focusing both in silico and in solution‐study on the structural and functional features of individual enzymatic variants in light to understand the genotype to phenotype correlation and to set up appropriate precision medicine treatments. Several AADC pathogenic variants present both in homozygosis (Bisello et al., 2022; Montioli et al., 2014; Montioli et al., 2016; Montioli et al., 2020) and compound heterozygosis (Longo et al., 2021; Montioli et al., 2018; Montioli et al., 2019; Rossignoli et al., 2021) have been cloned, expressed and purified. Overall, it was suggested that, in addition to local changes, also long‐range effects exerted by substituted amino acids present in AADC variants mapping far from the active site could be responsible for decreased enzyme function (Montioli et al., 2020). For defining the extent of the loss‐of‐function, related to disease severity, it would be important to characterize the protein structural regions more contributing to the drop of catalytic activity. The crystal structure of the mammalian pig AADC enzyme is dated 2001 (Burkhard et al., 2001) and, given the high sequence identity with the human counterpart (89%), it has represented a reliable model. Ten years later, the solved structure of the human apoAADC showed an open dimer with the subunits moved 20 Å apart from each other and the active sites totally solvent exposed (Giardina et al., 2011). This was interpreted in terms of different conformations occurring from the open apo to the closed holo state (Giardina et al., 2011; Paiardini et al., 2017) upon PLP binding. In all the released apo and holo structures, the catalytic loop (CL), containing the catalytic Tyr332 (Bertoldi et al., 2002), is not detectable given its high flexibility.

The availability of the human apoenzyme and the urgency to map pathogenic amino acid substitutions on a human structure, pushed also by the exponential increase of the number of newly identified variants (Bisello & Bertoldi, 2022), prompted us to solve the three‐dimensional (3D) human holo structure at physiological pH in the absence and in the presence of L‐Dopa methylester (DME) an esterified Dopa analog (Moore et al., 1997) commercially known as melevodopa and used in PD as a source of L‐Dopa in combination with the inhibitor carbidopa (Juncos et al., 1987). Here, we have obtained, for the first time, the structure of the external aldimine of human AADC complexed with DME with the definition of the electron density map for the CL, highlighting how this element is grasped to all protein domains, all involved in the stabilization of the catalytic competent complex. In addition, by means of a multiscale cooperative approach spanning from crystallographic analyses to all‐atoms and coarse‐grained (CG) molecular dynamics (MD) simulations of apo and holoAADC, limited proteolysis experiments coupled to mass spectrometry and small angle x‐ray scattering (SAXS), we explored mobility and flexibility of the homodimeric protein and determined the molecular geometry of the molecule in solution. Finally, we interpreted the kinetic parameters of AADC deficiency variants mapping both at the active site and distant from the active site but connected to it in terms of structural dynamics. The knowledge of the affected structural regions could allow to predict the extent of severity in AADC deficiency.

## 2 RESULTS AND DISCUSSION

### 2.1 CL of human AADC is trapped in the external aldimine intermediate and networked to residues of all protein domains

The crystal structure of human holoAADC in the presence of the substrate analog DME (Table S1) shows (for the first time) electron density of the whole polypeptide chain, including the CL (amino acids 327–341), highly disordered in the native form, that here is trapped in the external aldimine conformation (Supporting Information Results and Discussion and Figures S1 and S2).

**FIGURE 1:**
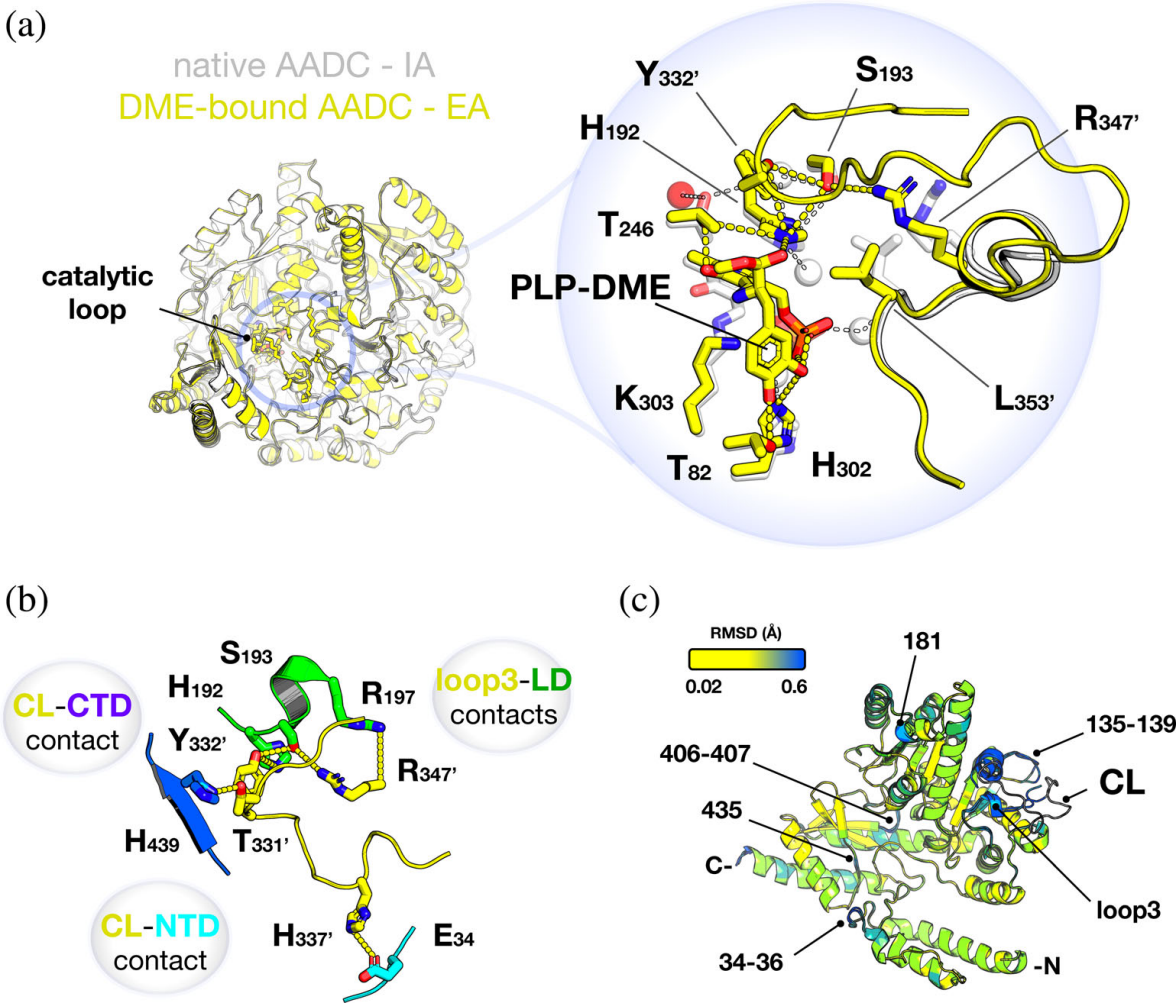
Crystal structure of human holoAADC in its internal (IA) and external aldimine (EA) forms. (a) Alignment of dimeric native AADC (white) and DME‐bound AADC (yellow). The CL of one subunit is shown. CL residues and the PLP‐DME external aldimine are represented as sticks in the zoom. Structural water molecules are white spheres in native AADC and red spheres in the DME‐bound AADC. Polar weak interactions are shown with white and yellow dashed lines for native and DME‐bound AADC, respectively. The prime denotes residues belonging to the neighboring subunit. (b) Network of interactions established between the CL and the opposite active site residues belonging to different protein domains: NTD in cyan, LD in green and CTD in blue. For clearness, only the segment involved in interactions with CL of each domain is represented as cartoon. (c) Superposition of native and DME‐bound monomeric AADC colored by RMSD. The CL (present only in DME‐bound AADC) is colored gray.

The superposition of the active sites of human native and DME‐bound AADC shows that the pyridine ring of PLP faintly tilts when it converts from the internal to the external aldimine, being the main difference associated to the presence of the CL from the neighboring subunit orienting the catalytic Tyr332' (the prime denotes residues belonging to the neighboring subunit) in a H‐bond relay with His192 and Ser193 in the proximity to the a‐C of the ligand (Figure 1a). Slight changes are observed in the orientation of loop3 residues Arg347' and Leu353' contributing *in trans* to the active site in the transition from internal to external aldimine. The presence of the CL in the PLP‐DME conformation plugs the active site, displacing crystallographic water molecules and allowing the catechol side chain of DME to be held in place by hydrophobic and hydrophilic interactions established by several residues. In addition, the hooking of the CL is assisted by a network of weak intersubunit interactions (Figure 1b): Thr331' is H‐bonded to the imidazole ring of His439 belonging to the sheet β10 (residues 436–440) of the C‐terminal domain (CTD) and His337' contacts Glu34 (belonging to the loop region connecting helix α1 to helix α2) of the N‐terminal domain (NTD). Residues of loop3 are also engaged in networking the CL to all protein domains (Figure S2H).

Besides these local changes, further small alterations far from the active site are observed by monitoring the root mean square deviation (RMSD) between native and DME‐bound AADC structures. The highest deviations are observed for loop3 (since it is solved only in the DME‐bound AADC). Slight changes are also visible in the backbone of residues 34–36 belonging to the α1‐loop‐α2 motif (NTD), in the stretch containing residues 434–436, immediately after helix α16 (417–430) and participating to sheet β10 (436–440) (CTD), as well as in the segment 135–139 (Figure 1c). Thus, positioning of the CL at the active site establishes a large network of interactions, extends the contacts among subunits and contributes to long‐range small movements of some protein regions. In addition, the structure complexed with DME presents some slight changes, with respect to the native one and to previously solved structures (Burkhard et al., 2001; Giardina et al., 2011), not only limited to the active site but also to regions far from it. Further details are reported in Supporting Information Results and Discussion, Figures S3 and S4.

### 2.2 AADC has an asymmetric elongated shape in solution

**FIGURE 2:**
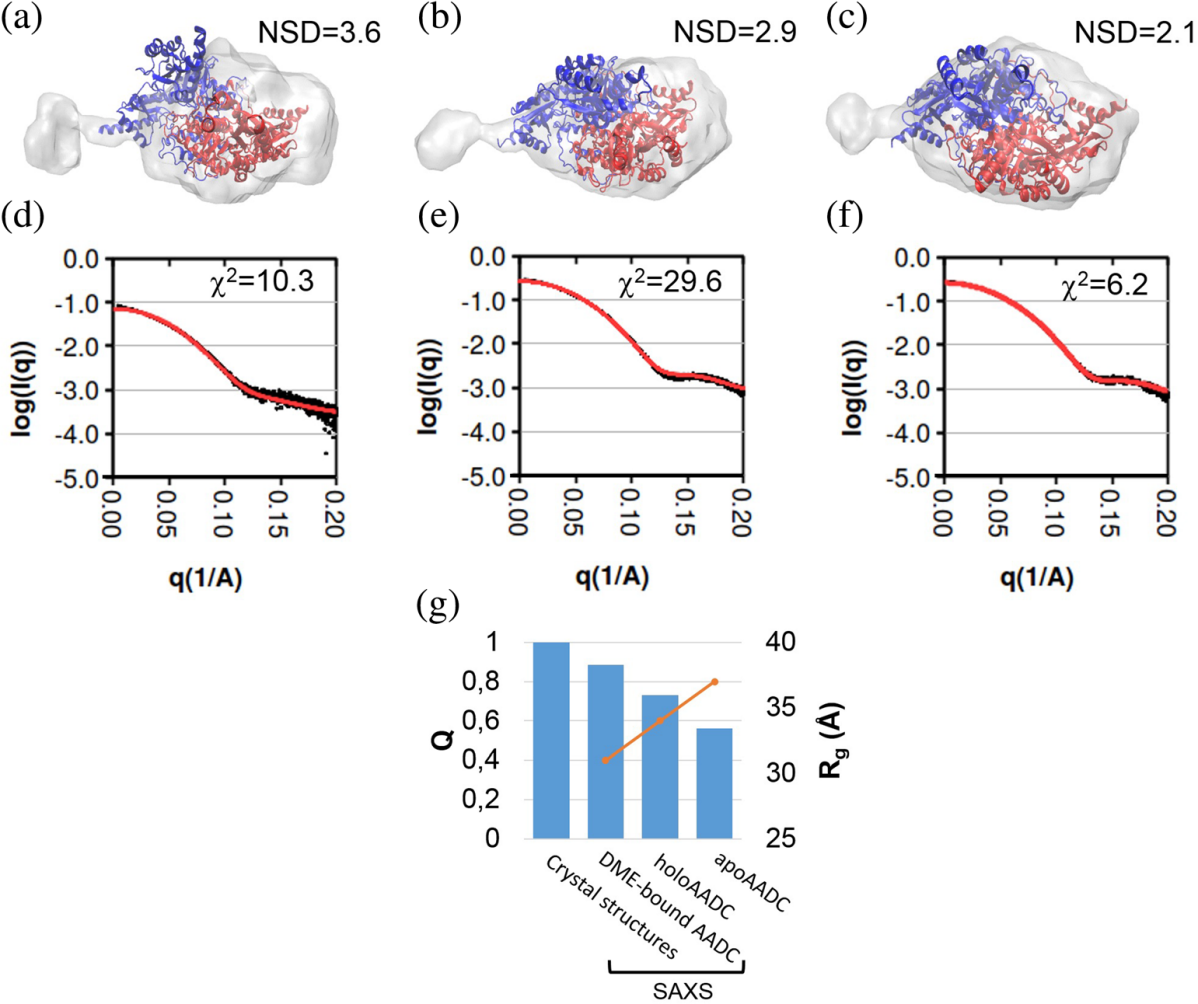
Modeling of SEC‐SAXS data and comparative analysis of the geometrical features of the AADC structural models. Top: structural models refined by flexible‐fitting molecular dynamics (cartoon representation, with the two chains in blue and red) superposed to the molecular envelope calculated from SAXS data (transparent surface) for apo (a), holo (b) and DME‐bound (c) AADC. The normalized spatial discrepancy (NSD) between the model and the envelope is reported. Bottom (d), (e), and (f): best fits of the same models with SAXS profiles. Profiles calculated from the model are shown in red line. The *χ* ^2^ of the fit is reported. (g) Geometrical features of the SAXS‐derived structural models. Quality of alignment (*Q*) between A and B chains of the AADC dimer (bars, axis on the left) compared its size, measured by the radius of gyration *R*_g_ determined by SAXS data.

Solution‐scattering data collected by SAXS coupled with size exclusion chromatography (SEC‐SAXS) analyses of human apoAADC, native holoAADC and DME‐bound AADC have been carried out in order to compare structural data acquired in solution with the crystal structures and possibly to reveal differences among them. The SEC‐SAXS geometrical characterization highlights a highly asymmetric shape of the experimental molecular envelope, and a large difference between the SAXS profiles of the apo and holo forms (Figure 2a–f). Modeling of SAXS data, performed by making flexible crystal structures through a refinement procedure restrained in both direct space (using the molecular envelope) and reciprocal space (using the SAXS profile), produced putative models of AADC in solution (shown superposed to the molecular envelope in Figures 2a–f and S5). The degree of symmetry of the structural models, as measured by the quality of the alignment between the two chains of the dimer, decreases for AADC in solution (Figure 2g). In fact, while in the crystal structures the two AADC chains are perfectly aligned (*Q* = 1) to comply with crystallographic symmetry, in the SAXS‐derived structures an asymmetry arises, which is larger for apo than for holo and it is further reduced in the presence of DME. Noteworthy, the size of the model measured by experimentally derived radius of gyration (*R*_g_) (Figure 2g) has the same trend as that shown by the asymmetry, with apo structure larger than holo one, and DME further reducing the holo size. The same trend is exhibited by other geometrical parameters derived by SEC‐SAXS data given in Table S2, such as the maximum interparticle distance (*D*_max_) and the Porod volume of the scattering object. Overall, all investigated species are dimers but the structures refined against SAXS data differ significantly from the respective crystal structures. In addition, a comparative analysis among AADC crystals and solution structures, performed by means of PCA applied to geometrical descriptors, shows a trend in the differences from apo to holo and to DME‐bound AADC (see Supporting Information Results and Discussion and Figures S5–S6). More specifically, apo and holo structures differ: (i) in the opening of the apodimer, as already evidenced by crystallography due to a relative hinge motion of apo chains in the LD region and interface opening (Giardina et al., 2011), (ii) in the extension towards the CTD not shown by crystallography and that SAXS suggests to be more pronounced for apo than for holo, that, in turn, is more extended than the DME‐bound AADC model where the distance between CTD is reduced. This longitudinal elongation breaks the symmetry of the dimer, giving rise to the observed asymmetry shown in Figure 2a–f. Interestingly, previous DLS measurements agree with SAXS data, thus corroborating the more compact structure of the holoAADC with respect to apoAADC (Bisello et al., 2022).

In summary, both apo and holoAADC in solution adopt a loose and highly asymmetric conformation extended on the longitudinal axis towards the CTD at the edge with the NTD. This elongation, that is not related to the open interface displayed by the apo structure (Giardina et al., 2011), seems to be more pronounced in the apo than in the holo structure. The presence of DME bound to PLP seems to compact the structure, maybe by stiffening AADC mobility while trapping CL. Notably, a discrepancy between the crystal structure and the asymmetric molecular shape in solution is also reported for the homologous mouse holo acidic amino acid decarboxylase GADL1 (23.3% sequence identity with AADC) whose loose conformation was suggested to be relevant for ligand binding and catalysis (Raasakka et al., 2018). In addition, while the SAXS data for holoGAD‐65 are in good agreement with the crystal structure for the holo form, those for the apo enzyme diverge and have been interpreted as a conformational ensemble of species characterizing this form (Kass et al., 2014).

Given the discrepancy among solved crystal structure and solution shape of both apo and holoAADC, we carried out limited proteolysis experiments, as assessed approach for probing structural changes (Fontana et al., 2004), in order to detect regions both exposed to the solvent and inherently flexible.

### 2.3 Limited proteolysis reveals different flexibility at the edge between NTD and CTD of human apo and holoAADC

The flexibility of CL is witnessed by its already known susceptibility to trypsin digestion at the Lys334–His335 peptide bond giving rise to conversion of the full‐length 54.7 kDa species into two fragments of 36.6 and 18.1 kDa (Bertoldi et al., 1999). The kinetic investigation carried out here evidences a faster disappearance of the 54.7 kDa band of apoAADC with respect to holoAADC, following trypsin digestion, that cannot be attributed to a different exposure of the CL stretch in the two species (Figure S7A,B), since, apoAADC exhibits an additional N‐terminal tryptic site (Arg27–Gln28), never identified before, that appears concomitantly to CL cleavage (Figure S7A and Table S3). Accordingly, the proteolytic patterns of holo and apoAADC could be represented as shown in Figure S7C.

Crystallographic data of human apo and holoAADC show that Arg27 is placed at the end of helix α1 and interacts with Glu61 of helix α3 through the formation of a salt‐bridge (Figure S7D). This saline interaction could favor the assembly of the NTD and in the meanwhile it could properly orient the stretch of the residues 28–36 towards elements of the CTD (the nearby helix α16 close to sheet β10), in turn connected to the active site. Gln28 interacts with Asp32 which is engaged with Lys431 of the CTD, thus reinforcing the network of interactions between NTD and CTD (Figure S7D). Remarkably, the nearby NTD (amino acids 32‐34) are also engaged in contacts with the CL when it is locked at the active site in the DME‐bound species, as reported above. Thus, a small displacement in NTD could be responsible for long‐range effects affecting CTD and the active site. Overall, proteolytic data imply that the edge between NTD and CTD in apoAADC is flexible, in agreement with the more loosened apoAADC SAXS model.

Altogether, limited proteolysis data (for more details see SI Results and Discussion, Figures S8, S9, Tables S4, S5) witness that holo and apoAADC have different flexibility at the above identified region of the NTD being this NTD stretch protected from digestion upon PLP binding. The structural alteration induced by apo‐to‐holo conformational change is thus reflected by modifications occurring at the NTD. On the contrary, CL flexibility is minimally affected by PLP binding since it is similarly digested in both apo and holo conformations. Instead, when the external aldimine with DME is formed, CL is mainly protected from digestion (Bisello et al., 2022; Montioli et al., 2020) (Figure S7A,B), being sheltered into the opposite active site by means of multiple connections mainly with CTD and NTD, as observed in the holoAADC‐DME crystal structure. While data collected by SAXS analyses corroborate the CTD‐NTD edge stretching, more evident in apo than in holoAADC, the Arg27–Gln28 peptide bond different exposure could not be revealed by the inspection of the crystal structures of human apo (Giardina et al., 2011) and holoAADC. Since the crystal structure is a static representation that cannot disclose the dynamic nature of CL and of entire regions of the protein essential for function, we have carried out all‐atoms MD simulations to investigate how mobility of AADC is linked to CL flexibility and possible NTD‐CTD different fluctuations in apo and holo enzyme conformations.

### 2.4 All‐atom MD simulations reveal flexibility of both polypeptide chains and asymmetry in apo and holoAADC

**FIGURE 3:**
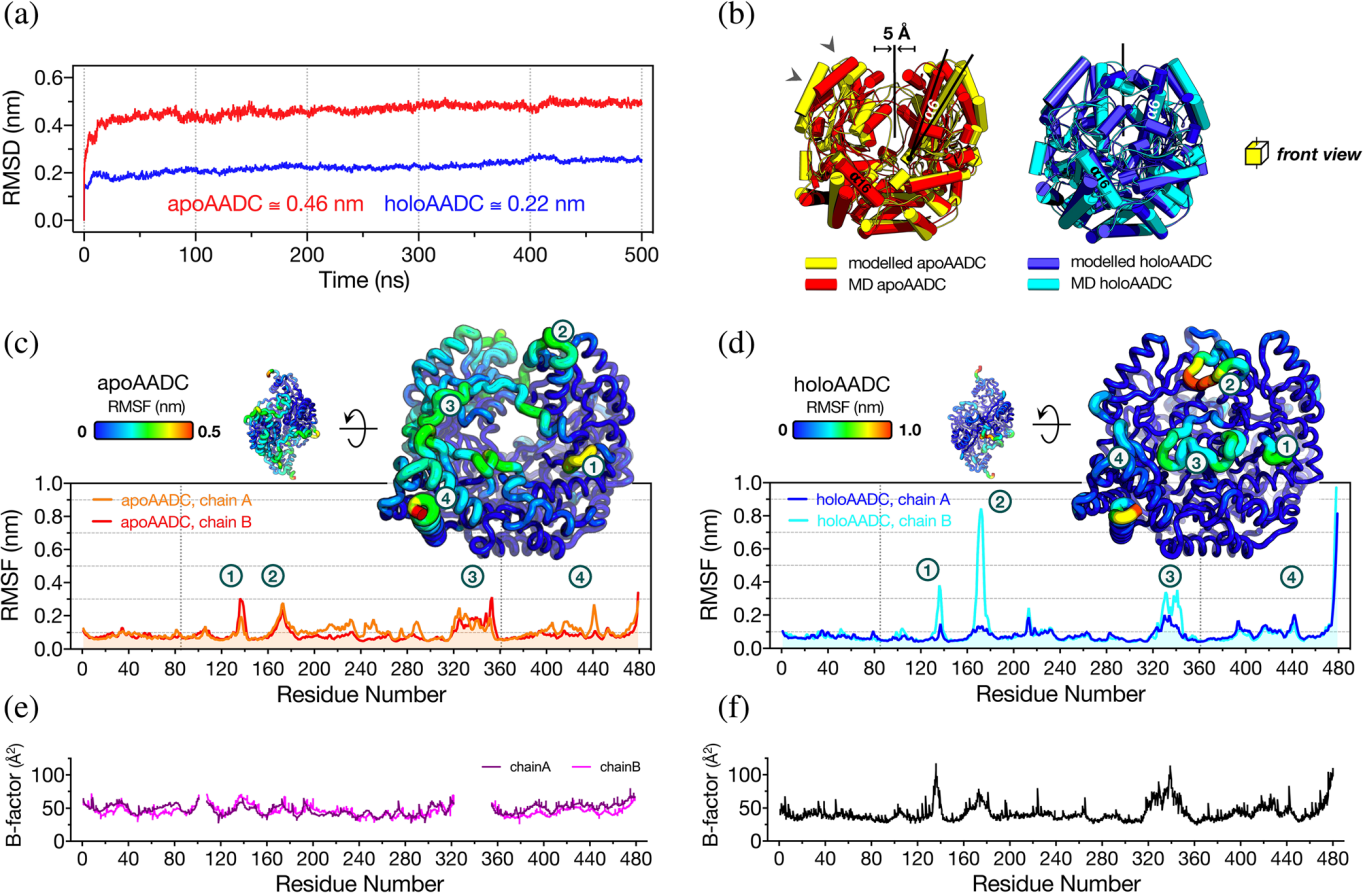
Structural rearrangement and flexibility of apo and holoAADC during molecular dynamics simulation. (a) Backbone RMSD as function of time for representative trajectories of dimeric apoAADC (red) and holoAADC (blue). (b) Comparison between the initial apo (yellow, PDB code: 3RBL) and holo (blue, PDB code: 8OR9) AADC and the structures equilibrated (red and cyan respectively) after 500 ns. Arrows indicates relaxations from initial to last MD snapshot. RMSF profile of (c) apoAADC and (d) holoAADC. Regions with highest flexibility are marked 1 to 4 both in the plots and in the all‐atom MD structural models. (e, f) crystallographic B‐factors for apo and holo amino acid chains taken from the crystal structures of apoAADC (PDB code: 3RBL) (Giardina et al., 2011) and holoAADC (PDB code: 8ORA, this work). The missing segments in the apo B‐factor profiles are due to missing electron density for those polypeptide chains (Giardina et al., 2011).

All‐atom MD simulations have been performed for 500 ns using apoAADC (Giardina et al., 2011) and the newly obtained native human holo coordinates (PDB code: 8OR9). As CL was not captured in these structures, it was modeled for both of them, as described in the Materials and Methods section. Inspection of the backbone RMSD along the simulations, indicates that both systems reach equilibrium after 50 ns of simulations (Figure 3a). In particular, the apo system reaches a plateau at 0.462 ± 0.001 nm, diverging from the initial configuration more than the holo counterpart that reaches stability at 0.220 ± 0.002 nm. As shown in Figure 3b, the LDs of the relaxed apoAADC are closer to each other with respect to the crystal structure (Bertoldi et al., 2008; Giardina et al., 2011; Paiardini et al., 2017), in an arrangement that is similar to that obtained by MD simulations with the homologous GAD (Kass et al., 2014). In depth, the divergence seems to be more prominent in one monomer, in a region around the apical part of the protein in proximity to helix α6 (residues 147–171) that moves apart by ~10 Å during the MD simulations with respect to the crystal structure. This helix has been already proposed to constitute a highly flexible and PLP‐sensitive region in the homologous human HDC and GAD (Kass et al., 2014; Rossignoli et al., 2018). As for the holo form, the equilibrated structure does not show important divergence in comparison to the crystal structure, even if some small changes are visible in close proximity to helix α6, as in the apo structure (Figure 3b). The calculation of the root mean square fluctuation (RMSF) shows that the utmost flexible regions of apo and holoAADC are very similar even if the two species exhibit a different profile of fluctuations. The highest values of the RMSF are observed for both species in the LD (in the region including residues 200–300, apoAADC exhibits higher values and small differences in flexibility between monomers), with peaks in proximity to segment 135–139 (this stretch has been already mentioned for its slight change in position in the crystal structures of AADC without and with DME), helix α6, loop3 as well as the CTD (helix α16 and sheet β10). Fluctuations spikes are present in holoAADC where one chain is more flexible than the other (Figure 3c,d). Notably, NTD exhibit low flexibility in both structures and the solvent accessible surface area of Arg27 in all‐atom MD analyses is invariant in all chains in both apo and holo forms (Figure S10A), even if its bond distance to Glu61 seems to oscillate asymmetrically in apoAADC until its breakage is captured by the last 20 ns of the simulation (Figure S10B, and Movie S1). Interestingly, the stretches showing the highest flexibility present highest crystallographic B‐factors (Figure 3e,f).

A closer inspection of the catalytically relevant element CL at the active site of both equilibrated apo and holoAADC indicates that AADC is a flexible protein. The position of the CL inside or outside the opposite active site triggers a reorganization not limited to the PLP pocket but also to the regions around it, supporting the modifications induced by CL closure upon DME binding through contacts to all protein domains. An asymmetry is also visible looking at the volume occupied by an extended surface (defining a sort of internal cavity) between the two active sites of holoAADC shaped by an extensive network of connections. In addition, CL dynamics seems to be related not only to the presence of the coenzyme, but also to contact networks at the NTD‐CTD edge that at the end of the simulation results highly asymmetric both for apo and holoAADC (for more details see SI Results and Discussion, Figures S10C, S11, S12, and Movie S2).

Altogether, the different flexibility of the NTD‐CTD edge in apo and holo species cannot be caught by the atomistic MD simulations, even if a difference in geometrical shape and an asymmetric, loosened, extended, and less compact structure for the apoAADC has been revealed by combination of all‐atoms MD simulations, SAXS analyses and limited proteolysis. In order to achieve a longer and large‐scale dynamics that could be relevant to assess the dynamical properties of the protein domains and/or conformational changes, we carried out CGMD simulations that enable evaluations of global motions.

### 2.5 CGMD simulations of AADC suggest asymmetry and mobility of the protein dimer

**FIGURE 4:**
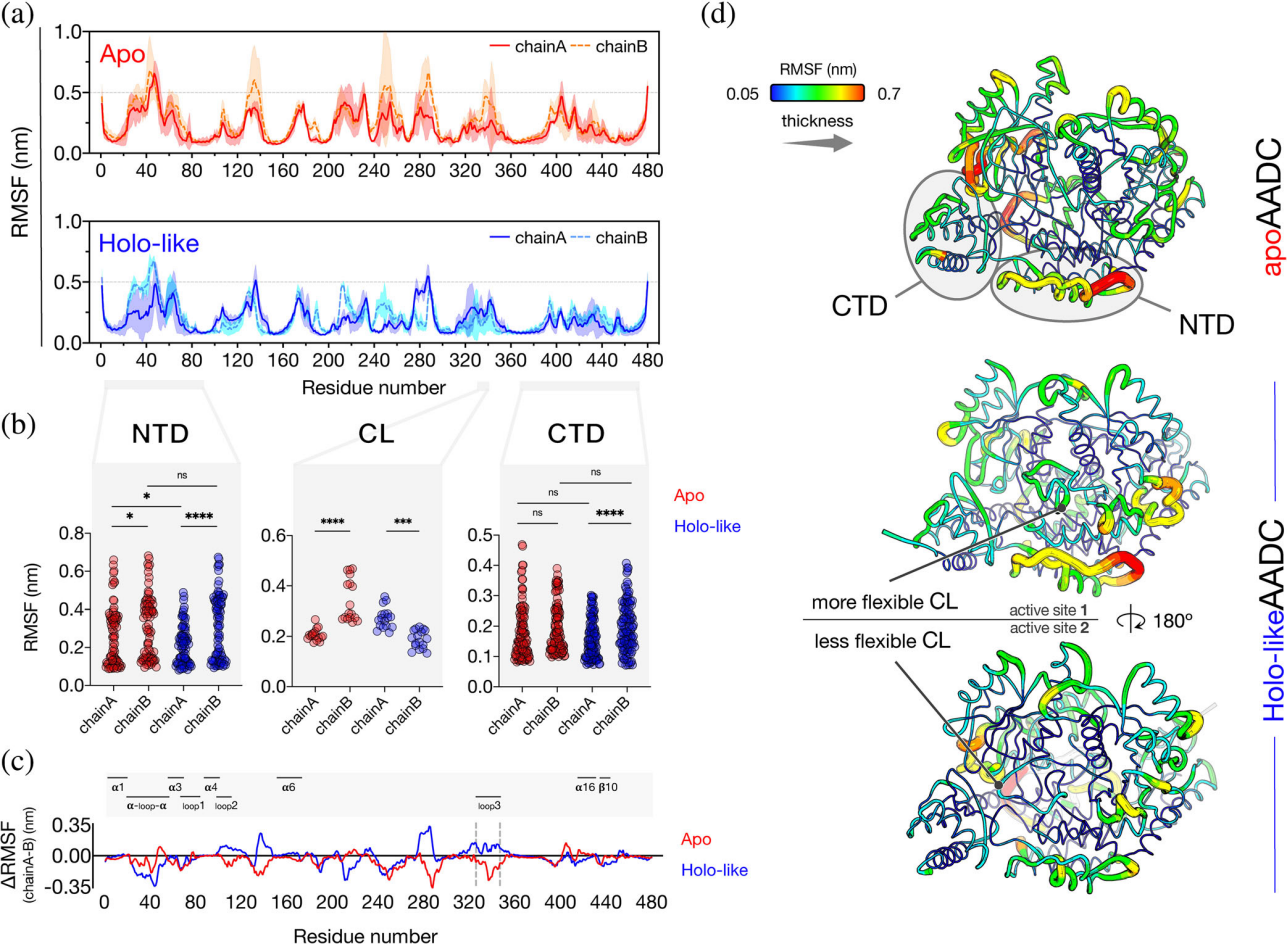
CGMD simulations of apo and holo‐like AADC models. (a) Calculated average RMSF value of the two monomers of each species as a function of residue number from three independent simulation runs (shaded area) of 10 μs. ApoAADC, straight and dashed red for the two monomers, holo‐likeAADC straight and dashed blue for the two monomers. (b) Plot of the values of averaged RMSF for NTD, CTD and loop3 in the enzymatic species; ns *p* > 0.05, *p ≤ 0.05, ***p ≤ 0.001, ****p ≤ 0.0001. (c) Averaged ΔRMSF as a function of residue number, apoAADC is red, holo‐likeAADC is colored blue. (d) Averaged RMSF values derived by CGMD simulations of apo and holo‐likeAADC were represented on the all‐atom MD apo and holoAADC models at 500 ns to show secondary structure elements.

CGMD simulations were carried out on human apoAADC and holo‐like AADC species (see Methods, Section 3) for a total of 20 μs. All calculations and analyses were based on the equilibrated last 10 μs of each simulation. The RMSD of each simulation show that the global structure of AADC was maintained throughout the entire simulation time (Figure S13A). RMSF values reveal an overall asymmetric behavior between the two polypeptide chains of both apo and holo‐like species (Figure 4a). The fluctuations in NTD are significantly different between the two chains of both species, more in the holo‐like than in apoAADC. A difference in flexibility in this region is also displayed between chains A of both species, while chains B behave similarly (Figure 4b). RMSF values of residues belonging to the CL are significantly different between the chains of both apo and holo‐like species, while differences in the fluctuations of two CTD are evident only in the holo‐like AADC (Figure 4b). Thus, the flexibility is overall higher in the apoAADC, while the holo‐like species exhibits a more pronounced asymmetric profile of fluctuations (Figure 4c,d). Notably, considering the dimeric assembly of the holo‐like species, this leads to a less flexible CL facing a more rigid NTD‐CTD region on one side and a more flexible CL facing a less rigid NTD‐CTD on the other (Figure 4d).

The CGMD models (see Methods) of apo and holo‐like AADC reflect a marked asymmetry for both species (Figure S13B), in agreement with SAXS models. The 2D average distance maps indicate that the distance between NTD and CTD of each polypeptide chain in the dimeric apo and holo‐like species is different, revealing the asymmetry of each dimer and that, although asymmetric, these domains are closer to each other in holo‐like AADC (Figure S13C). In addition, the distance between the CTD of each subunit to loop3' of the neighboring subunit, is higher in apo than in the holo‐like species (for details see SI Results and Discussion and Figure S13C).

These data, in agreement with all‐atoms MD simulations, proteolysis experiments, features of variants of the proteolytic sites and SAXS analyses, corroborate that AADC possesses a pronounced asymmetry and its mobility at the NTD/CTD boundary seems to be related to modifications taking place at the active site. This is reminiscent of the interplay among CL and CTD in GAD (Kass et al., 2014; Langendorf et al., 2013). Thus, we hypothesized that the NTD‐CTD region might play a role in the structural dynamics of the protein and that its integrity is essential for catalysis.

### 2.6 Catalytic efficiency of AADC deficiency variants at the active site, at the interface and at the NTD/CTD edge is highly affected

**FIGURE 5:**
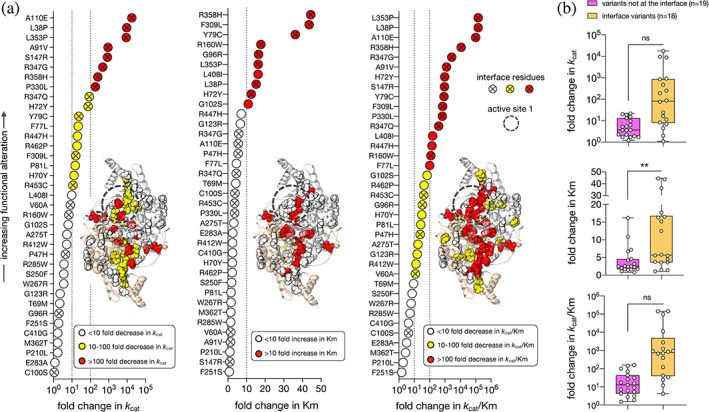
Change in *k*_cat_, *K*_m_ and *k*_cat_/*K*_m_ of AADC deficiency pathogenic variants mapping in all AADC domains. (a) The catalytic parameters *k*_cat_ and *K*_m_ as well as the catalytic efficiency *k*_cat_/*K*_m_ are represented as fold change with respect to the WT AADC. Color codes are reported in the figure. The symbol circle open denotes residues not belonging to the interface, a circle with a cross represent interface residues. Near to each plot, residues (with the same color code) are localized on the 3D structure (PDB code: 8ORA) in order to visualize their position on the protein. Parameters of variants L38P (Montioli et al., 2014), A110E and L353P (Montioli et al., 2020) that were below the detection limit of the assay procedure carried out in previous papers have been redetermined using the more sensitive method assay developed in (Bisello et al., 2022) and resulted as follows: *k*_cat_ = 0.00058 ± 0.00004 s^−1^ and *K*_m_ = 0.24 ± 0.03 mM for L38P; *k*_cat_ = 0.00031 ± 0.00001 s^−1^ and *K*_m_ = 0.09 ± 0.01 mM for A110E and *k*_cat_ = 0.00062 ± 0.00006 s^−1^ and *K*_m_ = 0.26 ± 0.03 mM for L353P. (b) The fold change is shown with regard to residues grouped on the basis of their belonging (yellow) or not (magenta) to the interface, ns *p* > 0.05, **p ≤ 0.01.

AADC deficiency homodimeric variants represent total or partial loss‐of‐function species whose pathogenicity was discussed until now basing on the structural knowledge of the pig crystal structure of the holo enzyme. In particular, two clusters of variants were identified: those belonging to loop1 and interpreted as impaired in the apo‐to‐holo transition (Giardina et al., 2011; Montioli et al., 2014) and some few catalytic variants mapping at loop2, loop3, or at NTD (Montioli et al., 2014; 2016) whose molecular basis was difficult to determine. Given the sudden increase in the number of identified AADC deficiency variants in the last 3–4 years (from about 100 in 2019 [Himmelreich et al., 2019] to 581 on April 17, 2023, http://www.biopku.org/pnddb/) and the fact that structural dynamics seems to play an essential role in AADC features, we examined the already published catalytic parameters of 37 AADC deficiency homodimeric variants (Table S6) (Longo et al., 2021; Montioli et al., 2011; Montioli et al., 2014; Montioli et al., 2016; Montioli et al., 2018; Montioli et al., 2019; Montioli et al., 2020). The substituted residues are placed along the entire amino acidic sequence and half of them belong to the interface (18 out of 37, about 49%). The worst drops in *k*_cat_ (values from 10 to above 100‐fold lower than WT) are exhibited by 18 variants mapping at NTD (6 out of 18), at loop3 (including the CL) (6 out of 18), at CTD (3 out of 18) and at the LD in proximity to the active site (3 out of 18). Interestingly, they are mainly located at the interface (13 out of 18). The other 19 AADC variants map mostly at the LD (63%) and only few of them belong to the interface (26%) (Figure 5a). Ten variants show a marked decrease in substrate affinity *K*_m_ (values from 10 to over 30‐fold higher than that of the WT) and almost all of them (80%) belong to the interface in the region comprising the two interconnected active sites. The catalytic efficiency (*k*_cat_ */K*_m_) combines both catalytic parameters and shows that all the highly affected variants (catalytic efficiency decreased by ≥100‐fold) are interfacial residues (13 out of 16 residues) belonging to NTD/CTD, loop3 (including CL) or are part of the active site resulting more affected than those in other protein domains. The interfacial surface of contact among subunits and active sites seems to play a role in governing affinity for the substrate (*K*_m_) since a significant difference between residues belonging to the interface, with respect to those far from it, is visible (Figure 5b). Despite the chemistry of the individual amino acid substitution, the worst catalytically affected AADC variants are located at the interface at/near the active site, but also in regions far from it, in particular to the region at the NTD in contact with the CTD.

### 2.7 Conclusions

By combining various high‐ and low‐resolution structural approaches, we determined that AADC is a mobile homodimer presenting asymmetric behavior among subunits, thus behaving as a conformational heterodimer. Indeed, apo and holoAADC do not only differ in the conformational change from the open apo form to the closed holo one, as previously suggested (Giardina et al., 2011), but also in terms of geometrical parameters (Table 1) and flexibility. We argue that AADC molecular profile is an effect of interrelated mobility at the NTD‐CTD edge with respect to CL flexibility and thus interdependent on efficient catalysis. AADC deficiency pathogenic variants can represent hotspots in regions relevant for structural dynamics. Interestingly, the worst catalytically affected pathogenic variants map not only, as expected, at the active site but also in a region far from the active site identified at the NTD‐CTD edge. Our analyses show that asymmetry of AADC is related to its flexibility at the NTD, in turn, is structurally connected to CTD. In addition, PLP binding triggers NTD‐CTD changes that seem related to CL mobility and finally to catalysis, as a domino effect. Following this view, loop1 variants (residues 66–84), whose pathogenicity was previously attributed to impaired apo‐to‐holo conformational change (Giardina et al., 2011), can now be interpreted as a consequence of NTD‐CTD conformational mobility which acts in concert with CL flexibility. Notably, the interface of the AADC dimer is a crucial element in triggering efficient function. Here, we notice that all interface variants are markedly affected, especially in affinity for L‐Dopa, and that the two active sites seem to be organized in a shared surface whose integrity should be preserved for catalysis.

**TABLE 1:** AADC dimensional parameters evaluated by different techniques.

	Diameter (nm) DLS^a^	Gyration radius (*R*_g_) (nm) x‐ray structures	*R*_g_ (nm) aaMD^d^	*R*_g_ (nm) CGMD^e^
apoAADC	9.98 ± 0.02	3.00^b^	2.96 ± 0.01	2.80 ± 0.02
holoAADC	9.61 ± 0.04	2.78^c^	2.85 ± 0.01	2.75 ± 0.02

^a^ Data are taken from (Bisello et al., 2022). Hydrodynamic diameter is reported as mean ± SEM.

^b^ Evaluated from (Giardina et al., 2011) (PDB code: 3RBL).

^c^ Evaluated from this work (PDB code 8OR9).

^d^ Data are obtained from the last 200 ns of simulation and are reported as mean ± SD.

^e^ Data are obtained from the last 10 μs of simulation and are reported as mean ± SD of the three replicas.

A relevant output of these results is the possibility to interpret loss‐of‐function variants of AADC deficiency. Since no clear genotype–phenotype correlation for AADC deficiency exists until now, prediction of pathogenicity based on the affected protein region could be useful in coupling it to patients genotypes and ultimately to clinical phenotype.

## 3 MATERIALS AND METHODS

### 3.1 Materials

PLP, L‐Dopa, dopamine, L‐Dopa methylester (DME), hydroxylamine hydrochloride, isopropyl‐β‐D thiogalactopyranoside (IPTG), phenylmethylsulfonyl fluoride (PMSF), trichoroacetic‐acid (TCA), trypsin, protease inhibitor cocktail (P8849), PEG200, HEPES‐HCl, and TRIS–HCl were purchased from Sigma.

### 3.2 Molecular graphics, bioinformatic tools, and statistics

Structural analysis, measurements and figures was carried out by using PyMOL 2.2.3 (The PyMOL Molecular Graphics 50 System, Version 2.0 Schrödinger, LLC). Evolutionary conservation analysis of AADC residues was carried out by the ConSurf server using the human AADC sequence as input data (id: p20711). A conservation score from 1 (variable residue) to 9 (highly conserved residue) has been attributed to each residue and displayed as spectrum using PyMOL. The electrostatic surfaces has been obtained for the native and DME bound AADC crystallographic structures after assigning the partial charges with PDB2PQR and using the Adaptive Poisson‐Boltzman Solver (APBS) Electrostatic Plugin for PyMOL. Buried surface areas, tunnel and pores have been detected with PDBePISA and with MOLEonline web server (Sehnal et al., 2013). Human AADC sequence (P20711) was aligned with human group II α‐decarboxylase sequences (HDC: P19113, CSAD: Q9Y600, GAD65: Q05329, GAD67: Q99259) retrieved from UniProt database and aligned using Multiple Sequence Alignment software CLUSTALW on ConSurf server (http://consurf.tau.ac.il).

The kinetic parameters for AADC deficiency variants for L‐Dopa published so far (Bisello et al., 2022; Longo et al., 2021; Montioli et al., 2011; Montioli et al., 2014; Montioli et al., 2016; Montioli et al., 2018; Montioli et al., 2019; Montioli et al., 2020; Rossignoli et al., 2021) are reported here as fold change with respect to the parameters calculated for the respective WT. Variants are clustered into two groups, those mapping at the dimer interface and all the others. The statistical analysis was performed with a unpaired t‐test analysis conducted using Prism, 8.4.0, (GraphPad) “**” Significance ns *p* > 0.05, *p ≤ 0.05, **p ≤ 0.01, ***p ≤ 0.001, ****p ≤ 0.0001.

### 3.3 Crystallization

The purified protein was used at a concentration of about 20 mg per ml for the initial screen of crystallization conditions. Molecular Dimensions Structure Screens and MemGold2*™* were employed at 20°C with the hanging‐drop vapor diffusion method, mixing 1 μL of the protein solution (human holoAADC 20 mg/mL in 50 mM Hepes pH 7.4) with the same volume of the precipitating solution, and equilibrating versus a volume of 0.3 mL of the latter in the reservoir. Small crystals were obtained under the preliminary conditions of 0.1 M Tris pH 8.5, 44% PEG 200; in order to obtain better crystals, we further optimized the conditions by changing the buffer, pH and increasing the amount of protein by mixing 1.5 μL of protein solution with 0.5 μL of reservoir. Best crystals were obtained in 0.1 M Hepes pH 7.5, 40% PEG 200. Diffraction quality crystals were obtained in about 4–5 days at 20°C.

Several trials were carried out to obtain crystals of holo human AADC with DME. Since AADC catalyzes a slow paracatalytic reaction with methyl‐esterified substrates (Bertoldi et al., 2005; Moore et al., 1997), a co‐crystallization of a solution containing enzyme and DME incubated for the long times required for growing crystals resulted unsuccessful. Crystals of holo human AADC saturated with DME were thus obtained by soaking holo human AADC crystals with a large excess of DME (5 mM) for different times (1, 2, and 3 h). The 3‐h time was chosen since it allows electronic density for the ligand at the binding site. After this time passed crystals were flash‐frozen in liquid nitrogen.

### 3.4 Data collection and processing

The diffraction data were collected from crystals frozen at 100°K after a brief immersion in a mixture of 80% of the mother liquor and 20% glycerol. The data of holo human AADC crystals were collected on the XRD2 beamline of the Elettra Synchrotron in Trieste, instead those referred to holo human AADC saturated with DME were collected on the ID23‐2 beamline of the ESRF in Grenoble. Data were indexed, integrated, and reduced using the programs MOSFLM (Battye et al., 2011) and Scala (Evans, 2006). The processed data were converted to structure factors using the program TRUNCATE from the CCP4 suite (Winn et al., 2011).

### 3.5 Structure determination and refinement

The high resolution structure of human holoAADC was solved using the molecular replacement method as implemented in the program Molrep in the suggested space group P6_1_22 (Vagin & Teplyakov, 2010). The search probe used was monomer A of the model available in PDB without LLP, PLP and solvent molecules, solved at 2.8 Å resolution (Giardina et al., 2011) (PDB entry 3RCH). The rotation function gave an unambiguous answer with a Rf/sigma coefficient of 8.31. The highest peak of the translation function had a Tf/sigma of 24.22, a score of 41.6 and an *R* factor of 48.5 for the data in the 70.9–1.9 Å resolution interval. Examination of the molecular packing in the unit cell after rigid body refinement showed that there is no clashing with the symmetry related molecules in this space group and confirmed that the search model was indeed properly oriented and positioned in the unit cell.

The other crystal form (saturated with DME) was isomorphous to the human holoAADC and therefore difference Fourier maps were calculated directly with the experimental data and the phases of the solved structure.

The initial models were first refined by simulated annealing, using the program Phenix.refine (Afonine et al., 2012; Davis et al., 2007), present in the suite Phenix (Adams et al., 2010). The final refinement of the two crystal forms of AADC was carried out by a series of several rounds of positional refinement alternated with manual model revisions using the program Coot (Emsley et al., 2010) and the refinement programs Refmac (Murshudov et al., 2011) and Phenix.refine.

During the process of refinement and model building the quality of the models was checked with MolProbity (Chen et al., 2010). TLS refinement has been used throughout the refinement process and the ligand molecules were modeled in the residual density identified in the Fo‐Fc and 2Fo‐Fc maps. Solvent molecules were added to the models in the final stages of refinement according to hydrogen‐bond criteria and only if their B factors refined to reasonable values and if they improved the R free.

The diffraction data and refinement statistics of all the models are summarized in Table S1.

### 3.6 Analysis of the models

The superposition of the models matching the secondary structure was performed using the subroutine SSM Superposition of the program Coot (Krissinel & Henrick, 2004). The distances between the ligand and protein atoms were calculated with the CCP4 program CONTACT (Tadeusz Skarzynski, Imperial College, London, 1.12.88). Figures of the structures were prepared and rendered with PyMOL (http://www.pymol.org).

### 3.7 Modeling of AADC and Lys303‐PLP parametrization

To obtain complete structures of the enzyme on the apo and holo states of the enzyme, we performed molecular modeling using the available crystal structures as templates. The apo state of the enzyme was modeled using the crystal structure of the human AADC protein on the same state (PDB code: 3RBL) using Modeler v9.21 (Webb & Sali, 2016). One hundred models for the apo structure were generated and assessed through DOPE (Shen & Sali, 2006) and GA341 (Melo et al., 2002) scores. The best model (presenting both CL exposed to the solvent) was chosen according these scores and visual observation to avoid crossing loops. The same modeling protocol was applied to model the holo state of the enzyme against the crystal structure on the same state (PDB code: 8OR9). The loops were modeled using Swissmodel (Waterhouse et al., 2018). Once the holo state was modeled, the Lysine‐PLP was manually introduced in the structure on position 303 of both chains by optimal superimposition with the templates and then by transferring the coordinates. Topology parameters of the zwitterionic form of Lys303‐PLP were generated using antechamber package of the AMBER20 tools (Case DA, 2020). The atomic partial charges were computed according the bcc scheme (Jakalian et al., 2002) and the AMBER atom types were assigned. Then, the residue library and parameters forcefield were prepared according the ff99SB force field. Finally, the topology files were converted to GROMACS' compatible files using the ACPYPE tool (Sousa da Silva & Vranken, 2012).

### 3.8 MD simulations

All atom MD simulations of the apo and holo states of the enzyme were carried out using the GROMACS program, version 2019 (Van Der Spoel et al., 2005). For the holo state simulation, since the Lys303‐PLP parameters are not included by default in the most commonly used force‐fields, they had to be included manually as described above. Water, described with the TIP3P water model (Jorgensen et al., 1983), and ion molecules (0.154 M of Na^+^/Cl^−^ to mimic physiological conditions) were added to solvate the systems. Each system was then equilibrated through a complete workflow: steepest descents minimization of 5000 steps, NVT equilibration of 100 ps, NPT equilibration of 100 ps, and MD production under the NPT ensemble for 500 ns. Simulations were carried out with an integration time‐step of 2 fs with a coupled temperature of 300 K using the V‐rescale thermostat (Bussi et al., 2007) and a coupled pressured of 1 bar using the Parrinello‐Rahman barostat (Parrinello & Rahman, 1981). The best holo model presents one CL loop open and the other closed on the homodimeric AADC.

For the CGMD simulations, the Martini forcefield v.2.2 was used (Monticelli et al., 2008). The coarse‐grained protein coordinates were generated converting the all‐atom models with the martinize.py script (de Jong et al., 2013). Due to the technical difficulties of generating the Martini CG parameters for the Lysine‐PLP, we have run an apo simulation starting from an holo conformation. By measuring the distances between the centers of mass of Lys303 and Leu353 we inferred that this apo structure closely mimics the holo conformation. As a result, we refer to this structure as “holo‐like”. Each CG system was equilibrated by funneling the system though the workflow: steepest descents minimization of 5000 steps, NVT equilibration of 20 ns, NPT equilibration of 20 ns, and MD production under the NPT ensemble for 20 μs. The CG simulations were carried out with an integration time step of 20 fs with a coupled temperature of 300 K using the V‐rescale thermostat (Bussi et al., 2007) and a coupled pressure of 1 bar using the Parrinello‐Rahman barostat (Parrinello & Rahman, 1981). Cluster analysis was performed on each system using the “cluster” tool from the GROMACS package, applying the GROMOS (Daura et al., 1999) algorithm with a cut‐off of 50 Å.

All calculations were performed within a GPU node available by the computational platform from the “Centro Piattaforme Tecnologiche” of the University of Verona.

### 3.9 SAXS measurements and data analysis, modeling and structure comparison

Measurements of SAXS coupled with in‐line size‐exclusion chromatography (SEC) were performed at the beamline B21 of the Diamond Light Source (Didcot, UK), equipped with a EIGER 4M detector (Dectris). Protein samples were concentrated up to 4 mg/mL. SEC‐SAXS data collections were performed at 20°C, by loading 50 μL of sample onto a 4.6 mL High Performance Shodex KW‐403 chromatographic column (10–700 kDa MW resolution range) connected to an Agilent 1200 HPLC system (Waters) and equilibrated with the same buffer of the protein sample. For such measurements, the integration time per frame was set to 3 s and data were collected up to a momentum transfer (*q*) of 0.34 Å^−1^.

Raw SAXS 2‐D images were processed by the DAWN processing pipeline (Filik et al., 2017) to produce normalized and radially integrated SAXS curves. SEC data and frame selection were performed by CHROMIXS (Panjkovich & Svergun, 2018) while averaging, background subtraction, and Guinier analysis were performed by PRIMUS (Konarev et al., 2003). The FIND_Dmax tool of SCATTER (ScAtter, 2017) was used to estimate the best value of the maximum momentum transfer *q* value (*q*_max_) to be used in data analysis.

The particle distance distribution function *P*(*r*) was determined by using GNOME (Semenyuk & Svergun, 1991) in the *q* range from the begin of the Guinier region to *q*_max_. The degree of ambiguity expected for the ab initio reconstruction of each dataset has been assessed by using AMBIMETER program (Petoukhov & Svergun, 2015). Ab initio molecular envelope determination were performed by generating 100 models of molecular envelope for each dataset by using the fast‐mode annealing procedure of DAMMIN program (Svergun, 1999). The first 20 models showing the best agreement with experimental data (lowest χ^2^) have been grouped by DAMCLUST (Petoukhov et al., 2012), by using a metrics based on the Normalized Spatial Discrepancy (NSD) (Kozin & Svergun, 2001), which tends to 0 for similar objects and exceeds 1 in the case of objects that differ each other. For each dataset, models of the most populated cluster have been superposed and merged by using DAMAVER (Volkov & Svergun, 2003) and the resulting model has been used as input for more accurate modeling (slow‐mode annealing procedure in DAMMIN (Svergun, 1999). An alternative molecular envelope calculation was carried out by using the program DENSS (Grant, 2018). The similarity between molecular envelopes has been assessed by the SUPCOMB program (Kozin & Svergun, 2001), which is based on the NSD distance metrics. Details about the SAXS data reduction and processing are given in Figure 2.

AADC structural models obtained by x‐ray diffraction or generated by MD were checked against SAXS data by using two figures of merits: *χ* ^2^ of the fit with the SAXS profile and NSD with the SAXS‐derived molecular envelope. The structural models that minimize both parameters have been taken as starting point for a molecular dynamics flexible fitting (MDFF) procedure (Trabuco et al., 2008), which implements the fitting of flexible atomic structures into a density map generated from the experimental molecular envelope. MDFF simulations were run as described in (Belviso et al., 2022), by using implicit solvent and a scaling factor of 0.1 kcal/mol for the MD potential associated with the SAXS envelope. Simulations were performed by using NAMD (NAnoscale Molecular Dynamics) (Phillips et al., 2020), and simulated data were analyzed by using Visual Molecular Dynamics (VMD) (Humphrey et al., 1996). MDFF simulations were monitored by calculating frame‐by‐frame the cross‐correlation coefficient (CORR) with the reference density map, the RMSD of the C_α_ atoms with respect to the initial structural model, and the *χ* ^2^ of the fit with the SAXS profile. The structural model with minimum *χ* ^2^ was taken as the best solution of the MDFF procedure. As validation of SAXS modeling, we observe that the models obtained by the MDFF procedure have an even better overlap with ab initio envelopes obtained by an independent calculation, based on the program DENSS (Figure S5). The best structural models refined by MDFF were analyzed by calculating selected geometric properties using PyMOL scripts (PyMOL Molecular Graphics System, Version 2.4 Schrödinger, LLC). Geometrical features from different models were compared using the Principal Component Analysis (PCA) and hierarchic clustering implemented in the program RootProf (Caliandro & Belviso, 2014). The asymmetry between the two AADC chains has been assessed using the program SUPERPOSE (Krissinel & Henrick, 2004) which evaluates the alignment quality:

(1) $$Q=\left(\frac{N_{\text{align}}^{2}}{N_{A}N_{B}}\right)\frac{1}{1+{\left(\frac{\text{RMSD}}{3}\right)}^{2}}$$

where *N*_align_ is the number of aligned residues, RMSD is the room mean square deviation of their C_α_ atoms, *N*_A_ and *N*_B_ are the number of residues in A and B chains of AADC. Q reaches 1 only for identical structures (*N*_align_ = *N*_A_ = *N*_B_ and RMSD = 0), and decreases to zero with decreasing similarity (increasing RMSD or/and decreasing *N*_align_).

The SAXS data and refined models for apo, holo and DME‐bound AADC have been deposited in the SASBDB database (Kikhney et al., 2020) on entry n. SASDR89, SASDR99, and SASDRA9, respectively.

## AUTHOR CONTRIBUTIONS

Giovanni Bisello and Mariarita Bertoldi designed research; Giovanni Bisello, Rui P. Ribeiro, Massimiliano Perduca, Benny Danilo Belviso, Patrizia Polverino de' Laureto, Alejandro Giorgetti, Rocco Caliandro performed research; Giovanni Bisello, Rui P. Ribeiro, Massimiliano Perduca, Benny Danilo Belviso, Patrizia Polverino de' Laureto, Alejandro Giorgetti, Rocco Caliandro, and Mariarita Bertoldi analyzed and interpreted data; Mariarita Bertoldi wrote the article.

## CONFLICT OF INTEREST STATEMENT

The authors declare no conflict of interest.

## Supporting information


**Movie S1.** Trajectory of a 500 ns simulation of holo and apoAADC in all‐atoms MD simulations. The two dimeric structures are displaced in a 2 grids panel, holoAADC on the left and apoAADC on the right. ChainA and chainB of each protein species are rendered with different colors. The movie shows the first rearrangement of the initial crystallographic structures and focus on the NTD‐CTD edge at about 450 ns of simulations and residues discussed in the text are shown. For visualization clarity, water molecules and ions are not shown. Image smoothing was performed with a window sized of 3 frames, which may have produced slight distortion of certain structures.

**Movie S2.** A simplified view of the two asymmetric holoAADC active sites showing the closed and open CLs and some active site elements (described in the text) are here displayed during the simulation time. Water molecules, ions and the other part of the protein are not shown. Image smoothing was performed with a window sized of 3 frames, which may have produced slight distortion of certain structures.

**Data S1.** Supporting Information.

## Data Availability

The x‐ray diffraction data and crystal structures of human holo native and DME‐bound AADC can be found in the PDB databank entry 8OR9, 8ORA, respectively. The SAXS data and the low‐resolution models of apo, holo and DME‐bound AADC can be found in the SASBDB databank entry n. SASDR89, SASDR99, and SASDRA9, respectively.
