# Supplementary material for: Human aromatic amino acid decarboxylase is an asymmetric and flexible enzyme: Implication in aromatic amino acid decarboxylase deficiency
Source: Protein Sci. 2023 Aug 1;32(8):e4732. doi: 10.1002/pro.4732 (PMC10382914; doi:10.1002/pro.4732)
Supplement: Supplementary file 3 — Data S1. Supporting Information. [file PRO-32-e4732-s002.docx]

Human aromatic amino acid decarboxylase is an asymmetric and flexible enzyme: implication in AADC deficiency.

Giovanni Bisello^1,a^, Rui P. Ribeiro^2,a^, Massimiliano Perduca^2,^* Benny Danilo Belviso^3^, Patrizia Polverino de’ Laureto^4^, Alejandro Giorgetti^2^, Rocco Caliandro^3^ and Mariarita Bertoldi^1,^*

^1^Department of Neuroscience, Biomedicine and Movement Sciences, Section of Biological Chemistry, Strada Le Grazie 8, 37134 Verona, Italy.

^2^Department of Biotechnology, University of Verona, Strada Le Grazie, 15, 37134 Verona, Italy.

^3^Institute of Crystallography, CNR, via Amendola 122/o, Bari 70126, Italy.

^4^Department of Pharmaceutical and Pharmacological Sciences, University of Padova, 35131 Padova, Italy.

^a^These authors contributed equally as co-first authors.

^*^To whom correspondence could be addressed: Mariarita Bertoldi

**Email:** mita.bertoldi@univr.it

Correspondence could be also addressed to: Massimilano Perduca

**Email:**  [massimiliano.perduca@univr.it](mailto:xxxxx@xxxx.xxx)

**This PDF file includes:**

Supporting text: Supplemental Results and Discussion and Supplemental Materials and Methods

Figures S1 to S13

Tables S1 to S6

Legends for Movies S1 to S2

SI References (23 references)

**Other supporting materials for this manuscript include the following:**

Movies S1 to S2

Supporting Information Text

**Supplementary Results and Discussion**

**Crystal structure of human holo AADC at physiological pH in the absence and presence of DME**

Human native holoAADC (space group P6_1_22), differently from the holo pig ^1^ (root mean square deviation (RMSD) ≈ 0.35 Å) and the apo human ^2^, is present as a single polypeptide chain in the asymmetric unit. It is composed by three domains: i) a N-terminal domain (NTD, residues 1-85) formed by three α-helices, making a hinge with the facing α-helices of the NTD belonging to the opposite subunit ^3^, and by an unstructured segment called loop1 (residues 66-84); ii) a large domain (LD, residues 86-360) containing the PLP-binding site and composed by the core β-barrel, several secondary structure elements and two unstructured segments: loop2 (residues 99-109) and loop3 (residues 323-357) (**Fig. S1A**). Both loops extend into the opposite active site and, specifically, loop2 concurs to the architecture of the substrate aromatic side chain pocket, while loop3 protrudes towards the opposite subunit but lacks of the flexible CL segment (residues 327-341) that contains the catalytic Tyr332 ^4^. Finally, iii) the C-terminal domain (CTD, residues 361-480) is topologically adjacent to both NTD and LD and extends like a wing in longitudinal direction (**Fig. S1A**). The functional compact, strictly interlocked, antisymmetric homodimer has been obtained applying the symmetry operators and confirms the global oligomeric arrangement of this group of α-decarboxylases ^3^ belonging to the Fold-type I of PLP-enzymes ^5,6^ (**Fig. S1B)**.

In its resting state, PLP (occupancy = 1) is bound as the internal aldimine species with Lys303 (**Fig. S2A**) and the active sites of the two subunits are located at the dimer interface in close proximity to each other with loop2 and loop3 of one subunit protruding into the PLP binding cleft of the other (**Fig. S2B**). As the pig enzyme ^1^, each active site is mainly composed by residues of the same subunit binding the cofactor and stabilizing it (**Fig. S2A,B**). In details, His192 and Ala273 stack with the pyridine ring, Asp271 makes a H-bond with the pyridine nitrogen keeping it protonated and assuring the electron withdrawing capacity of PLP, the Ser147-Ala148-Ser149 triad concurs in holding in place the phosphate group, Asn300 and His302 take place to the H-bonding network stabilizing the coenzyme into its pocket ^7^ as well as Thr246 that finds in proximity to the 3’O of the pyridine ring. The residues of loop1, Trp71, Tyr79, Phe80 and Thr82, together with Ile101’ and Phe103’ of loop2 (the prime denotes residues belonging to the other subunit), form a hydrophobic pocket suitable to accommodate the side chains of aromatic substrates. Several crystallographic water molecules (Wat a-f) are present at the active site concurring in H-bonding network with residues mentioned above (**Fig. S2A,B)**. The catalytically important loop3 does not show complete electron density since the CL is lacking (**Fig. S2C**). Nevertheless, it is evident how some residues of loop3 concur in building the active site architecture (**Fig. S2D)**: Asp345’ and His348’ are H-bonded to the guanidinium group of Arg347’, as suggested ^8^, and Arg197 binds the backbones of Arg347’, Gln350’ and Pro352’ to proper lock loop3 into the opposite active site. Through their backbone, Leu353’ and Gly354’contact a water molecule (wat e) that bridges the phosphate of PLP.

When DME binds to the aldehydic group of PLP (DME occupancy = 0.88 and PLP occupancy = 1) , it displaces Lys303 and forms the external aldimine intermediate (**Fig. S2E**) triggering an overall active site reorganization. The catechol group of the analog displaces water molecules and inserts into the hydrophobic pocket assembled by the aliphatic and aromatic residues mentioned above (**Fig. S2F**). The CL traps the external aldimine in a buried microenvironment and orients the catalytic Tyr332’ ^4^ towards the methylated carboxylate of DME by H-bonding both His192 at 4.5 Å and Ser193 at 3.7 Å (**Fig. S2F**). Electron density for the entire loop3 comprising the CL is present in this structure (**Fig. S2G**). The CL establishes contacts to both NTD and CTD (**Fig. S2*H***) and residues of loop3 additionally stabilize CL by creating several interactions: Asp329’ binds Asn338’, the carboxylate of Asp345’connects the imidazole of His335’, the guanidine group of Arg347’ moves to contact Ser193 that correctly orients Tyr332’ while its backbone is H-bonded to both His348’ and Arg197 of the LD (**Fig. S2H**). The formation of the external aldimine is further favored also by a positive surface electrostatic potential (**Fig. S2J).**

With respect to highly homologous PLP α-decarboxylases, the CL of human histidine decarboxylase (HDC, 52% sequence identity with AADC) complexed with histidine methylester ^9^ closes the active site providing a solvent-shielded environment. Despite the similar positioning, the catalytic Tyr334 (Tyr332 in AADC) was not reported to interact with other protein elements ^9^. Human glutamate decarboxylase-65 (GAD-65, 23% sequence identity with AADC) is highly disordered in its CL region, while in isoform-67 (GAD-67, 24% sequence identity with AADC), CL covers the opposite active site by directing Tyr434 (Tyr332 in AADC) next to the PLP-γ-aminobutyric acid product complex ^10^. Interestingly, the position of Tyr434 is different in the active site of the two monomers, underlining the extremely mobile feature of the CL, and no interactions of the CL with other protein elements are shown ^10^. However, we superposed the CL portion of these α-decarboxylases and observed a similar orientation of the CL with respect to the CTD and NTD regions also in HDC and GAD67, besides AADC (**Fig. S3A-C)**.

Notably, the interface of each AADC subunit extends from the NTD to the LD (**Fig. S4A**) covering a wide fraction of the solvent accessible area (27.3%), a value considerably higher than that (about 20%) of the prototypic Fold-type I aspartate aminotransferase ^11^. This becomes even higher in the DME-bound AADC (29.35%) due to the new contacts established by CL, as described above. Each active site finds in a pocket located at the interface and communicates to the opposite one through several residues (Ser147, Glu150, Phe309) and an intertwined network of water molecules in both the unliganded and DME-bound species. In details, in native AADC, Glu150 follows the Ser147-Ala148-Ser149 triad (anchoring the PLP phosphate) and connects (bridging Ser147) its active site to the neighboring one through a water molecule, in correspondence to the two-fold axis of the dimer. This molecule is hold in place by the opposite Glu150 approaching from the facing monomer. The water molecules fill the interface cavity and are further stabilized by interactions with Thr82 (loop1 of NTD) Ile101’ (loop2 of LD) and Arg356’ (loop3 of LD) as well as with Phe309 (LD) that projects inside the crevice as a gate (**Fig. S4B**). In the external aldimine conformation, the CL covers the facing active site as a lid, sealing the active sites from the solvent (**Fig. S4C**). Site-directed mutagenesis experiments of Glu150 (substituted to Ala and Gln) show that the loss of this residue inside the cleft affects affinity for both PLP (K_D_) and L-Dopa (K_m_) rather than catalytic constant (*k_cat_*), suggesting how this interface region could control cofactor and substrate binding (**Table S4**).

Notably, the structural relevance of the whole dimeric interface is highlighted by its high evolutionary conservation, not only at the active site, but extending to the entire surface of contact among subunits (**Fig. S4D**). This region is also characterized by lower crystallographic temperature factors (B-factors) suggesting a rigid core with respect to other more mobile parts, including the entire loop3 and the edge between NTD and CTD (**Fig.S4E**), since high values in crystallographic B-factor generally indicate a dynamic and thermal positional disorder ^12^.

**Comparative analysis of the geometrical features of the AADC structural models derived from SEC-SAXS experiments**

SEC-SAXS experiments of apo, holo and DME-bound AADC evidence not only a highly asymmetric molecular shape but also dissimilar features between the 3 species. The larger structural difference is visible between apo and holo native and DME-bound AADC models (it is captured by the first principal component (PC1), which explains 58.4% of the total data variance). Differences between holo structural models are instead captured by the second principal component (PC2), explaining 37.6% of the total data variance. It is worth noting that data projected along this component reproduce the same trend as found in the symmetry analysis. The analysis of the loadings allows to interpret the PCA results in terms of variations of geometrical features. We found that PC1 and PC2 describe structural movements of subunit A and B occurring in two orthogonal directions (**Fig.S6A-D**). Residues Glu172 and Leu470 of the same chain have been taken as geometrical descriptors of vertical and longitudinal extension of each subunit. Distance of Glu172 of one chain from Glu172 of the other can witness dimer opening in apo with respect to the holo structures. The PC1 strongly depends on the distance between the center of mass of the two chains A and B (descriptor 1), the distance between the α-carbon of the residues Glu173 of the two chains (descriptor 2), the angle formed between the residue Glu173 of the two chains and the center of mass of the dimer (descriptor 5), and the dihedral angle formed between the residues Glu173 and Leu470 of the two chains (descriptor 7). PC2 is dominated by the distance between the α-carbon of the residues Leu470 of the two chains (descriptor 3) and their angle with respect to the center of mass of the dimer (descriptor 6).

Notably, the fluctuating segment present only on one side of the envelope can be interpreted as one C-terminal His-tag of the purified recombinant AADC, corroborating the asymmetrical features of the AADC protein in solution.

**Limited proteolysis of WT apo and holoAADC**

The fact that the AADC CL is solvent exposed was already known by its susceptibility to trypsin digestion (Lys334-His335) ^13^ that leads to the conversion of the monomeric band of about 54 kDa into two fragments of about 36 kDa and 18 kDa ^13,14^ (**Fig. S7A**). These segments remain associated while the enzyme turns into a species unable to form dopamine ^13^, possibly for the mispositioning of the catalytic Tyr332 ^4^ and the impossibility to achieve a competent active site conformation ^15^. Here, we deepen the investigation to both apo and holo human AADC species, in order to assess if PLP influences the accessibility of the CL, given the fact that the CL is flexible in both apo and holo structure without ligand. We found that the 54.7 kDa apoAADC disappears with an initial rate higher (8.1 ± 0.8 min^-1^) than holoAADC (5.3 ± 0.7 min^-1^) (**Fig. S7B** and **Table S5**) but this could not be a sign of higher CL exposure in apoAADC since a concomitant tryptic cleavage occurs in this species at Arg27-Gln28 peptide bond giving rise to multiple species, in addition to the 36 kDa and 18 kDa, such as 51.5 kDa and 33.4 kDa, identified by mass spectrometry (**Fig. S7A,C** and **Table S3**). While holoAADC is cleaved only at CL, the rate of disappearance of the apo species is the sum of the two contributions (cleavage at CL and at NTD). However, we cannot easily attribute the rate of holo CL cleavage as an addend of the value measured for the apo since, given their different structure (^2^ and this work), the CL of the apoAADC could be differently exposed to trypsin.

A superposition of other human α-decarboxylases shows that Arg27 is conserved in the strictly homologous HDC, while other human α-decarboxylases present polar amino acids at the position of Arg27 and a Lys residue at the position of Gln28 of AADC. The latter residue is not conserved but its polar nature (responsible for interactions with the CTD) is retained since all other decarboxylases have a positive residue. In AADC, Arg27 is bonded to Glu61 of helix α3; this saline bridge is conserved only in HDC, while it is substituted by a polar or positive amino acid in the α−decarboxylases (**Fig. S7D,E**).

Interestingly, AADC proteolytic pattern differs from that of the homologous human GAD isoforms ^16^. While GAD-67 is resistant to proteolysis both in the apo and holo species, holoGAD-65 is cut only at the CL, as holoAADC. However, apoGAD-65 is cleaved at NTD (Lys49-Val50) as apoAADC (Lys49 of GAD-65 aligns to Gln28 of AADC, **Fig. S7D,E**), and, differently from apoAADC, is concomitantly cut at the LD and at CTD, but not at CL. This has been interpreted as a cofactor-dependent conformational change induced by the presence of PLP that governs flexibility of the CL and of the entire protein. In particular, a crosstalk between CL and CTD in the holo form was suggested by combination of proteolysis data with normal mode analysis ^16^. Intriguingly, CL in apoGAD-65 is not cleaved by trypsin ^16^, although it is supposed to remain flexible.

**Characterization of artificial AADC variants involved in the proteolytic sites**

In order to gain some insight into trypsinolysis of apo and holoAADC, we focused the investigation on residues involved in proteolytic sites (Arg27 and Lys334) or interacting with them (Glu61) and prepared and characterized R27A, R27Q, E61A and K334Q AADC recombinant species. All variants were obtained in good yields, comparable to that of the WT. At first, we expressed R27Q and K334Q variants to mutually abolish the tryptic sites maintaining a polar environment and a similar steric bulk. In terms of structural and functional effects of the amino acid substitutions, both apo and holo K334Q are minimally affected in both thermal stability since the Tm values at 222 nm (**Table S4)** are similar to those of the WT, and UV-visible dichroic spectra that are superposable to those of the WT (**Fig. S8A**). It also retains good catalytic competence with a catalytic efficiency that is 69% that of the WT and an identical affinity for PLP (**Table S4**). R27Q is instead more altered both in dichroic signals and Tm values as well as in affinity for PLP but retains a good, although decreased, catalytic efficiency reaching about 41% that of the WT (**Fig. S8A and Table S4)**. Being assured that these artificial amino acid substitutions do not dramatically impact AADC function, we performed limited tryptic proteolysis with holo and apo species of both variants to dissect the contribution of each proteolytic event. Notably, both apo and holoR27Q are cleaved only at the CL, as expected (**Fig. S8B**), with a rate of disappearance of the 54.7 kDa of about 7.8 ± 0.8 min^-1^ and 7.2 ± 0.6 min^-1^, respectively (**Fig. S8B** and **Table S5**), a value similar to that obtained with apoWT which, is instead cut at both CL and NTD. **Fig. S8C** compares initial rates of decrease of the full-length AADC for WT and different variants (see below).

While holoK334Q is trypsin-resistant as expected, apoK334Q is nicked only at the NTD with a rate of disappearance of the 54.7 kDa species of 3.7 ± 0.6 min^-1^ (**Table S5**), a value that if added to that of the holoWT, could account for two parallel cleavage events occurring in apoWT upon trypsin addition. Remarkably, some issues arise from these results. First, the CL is more flexible in apo/holo R27Q variant with respect to holo WT, since the rate of CL proteolysis in R27Q is higher. Thus, it can be argued that this amino acid substitution at the NTD exerts some effects on CL flexibility. Interestingly, the dichroic bands of the R27Q species, both in the near UV and in the visible region, are altered with respect to the WT, suggesting some conformational alterations (**Fig. S8A)**. Second, proteolysis carried out for longer times with apoK334Q shows the persistence of the native enzyme (54.7 kDa, amino acids 1-486) in addition to the lower molecular weight species (51.5 kDa, amino acids 28-486) due to NTD cleavage (**Fig. S8D)** with a first kinetic event of 1 hour leading to the accumulation of the 51.5 kDa up to 50% of relative amount and then gradually increasing up to 4 hours to about 55%. Notably, the 51.5 kDa band is a transient species for apoWT, and apo and holoE61A variant (see below), while it is the only species that accumulates in apoK334Q (**Fig. S8E**).

When apoK334Q is incubated in the presence of an excess of PLP, it regains almost full activity as the WT. Notably, previous apo digestion followed by subsequent reconstitution with PLP leads to a dimeric species that binds 2 mol of PLP, although with a bit lower affinity, being the measured equilibrium K_D_ for PLP equal to 154 ± 15 nM, increased by 1.5-fold (**Table S4**) and in a different microenvironment as shown by the altered tautomeric equilibrium of the internal aldimine species as witnessed by the 335 nm and 420 nm dichroic bands (**Fig S9A).** If apoWT is subjected to the same treatment, i.e. digested and then reconstituted with PLP, it binds the coenzyme with an equilibrium binding constant K_D_ of 186 ± 14 nM, increased by 1.8-fold (**Table S4**) and presents dichroic features similar to those of K334Q species (**Fig. S9A**). However, while apoWT digested and reloaded with PLP does not regain activity, apoK334Q treated under the same experimental conditions rescues about 50% activity (**Fig S9B).**

R27Q variant features deserve a particular explanation, since basically it is more structurally compromised even in the holo form (**Fig. S9A**). When apoR27Q is incubated with an excess of PLP it does regain about 30% of the original activity of holoR27Q (**Fig. S9B**). The coenzyme binds with an affinity about 5-fold decreased with respect to the WT (**Table S4**) and the dichroic spectrum evidences some structural alterations in the internal aldimine tautomeric equilibrium since the ratio 335 nm/420 nm is altered with respect to the WT (**Fig. S9A**). If apoR27Q is digested and then reconstituted with PLP, it is uncapable of regaining activity as the digested and reconstituted apoWT (**Fig. S9B**) while its equilibrium dissociation constant for the coenzyme, K_D_, remains almost unaltered with respect to the undigested apo species (K_D_ = 571 ± 38 nM).

Overall, these data underline that both NTD and CL are essential to enzyme function and suggest that when CL is not nicked, as in holoK334Q, the enzyme is fully functioning with a rate of nearly the same magnitude of the WT. ApoK334Q undergoes a trypsinolysis of the Arg27-Gln28 peptide bond to a 50% of the original enzyme content, that leads to a species that is 50% irreversibly inactivated. Even if an equilibrium between unnicked and nicked apoK334Q species could not be ruled out, the data are also evocative of a 50% Arg27-Gln28 peptide bond accessibility in the dimeric AADC due to a possible asymmetric arrangement in which one subunit is more susceptible than the other, revealing that NTD cleavage leads to incapability of recovering activity and suggesting that there is a structural link between NTD and the active site, resulting in a functional effect. Data collected with R27Q evidence how the NTD region integrity is essential to both structure and function and is related to the CL.

As a further step, the variants R27A and E61A have been prepared to monitor the effect of the breakage of the saline bridge involving Arg27. In particular, the substitution of either Arg27 or Glu61 with alanine abolishes any possible polar interaction, not guaranteed by the R27Q substitution. The proteolytic behavior of holo and apoR27A is similar to that of holo and apoR27Q (**Fig. S8B and S9B**), with a rate of disappearance of the 54.7 kDa species in apo and holo enzymes slightly faster than those of the corresponding R27Q forms (**Table S5**). Surprisingly, both apo and holoE61A show a fast decrease of the 54.7 kDa species (**Fig. S8B,D**), with a pattern displaying both CL and NTD breakages and accumulation of the 51.5 kDa species (**Fig. S8B)**, suggesting that even in the holoE61A variant, the Arg27-Gln28 region is more flexible. Both R27A and E61A exhibit structural and functional features similar to those of R27Q (**Table S4** and **Fig. S8A**). They display a decreased thermal stability and altered dichroic signals of their holo species as well as internal aldimine PLP tautomeric equilibrium with a prevalence of the ketoenamine tautomer. The equilibrium dissociation constant for PLP (K_D(PLP)_) is increased by 4.3 and 3-fold respectively (**Table S4**), similarly to R27Q. Catalytic efficiency is nearly 7% that of the WT for R27A similar to that (6%) for E61A (**Table S4**). Overall, the disruption of the saline bond between Arg27 and Glu61 affects AADC both structurally and functionally increasing the flexibility of the NTD-CTD edge in holoE61A. This corroborates the correlation between this region at NTD, in contact with CTD, and AADC activity, mediated by CL flexibility.

**All-atom MD simulations of modelled apo and holoAADC**

An inspection of the NTD-CTD contacts at the end of the MD simulations (500 ns) shows that intra-subunit network of interactions involving residues Glu25, Asp32, Gln28 (NTD) bridging His459, Gln462, and Lys431 (CTD) presents differences in the two monomers (more in the apo than in the holo enzyme) as well as the inter-subunit network established by NTD residues (Asp51, Asp55, Asp59) with CTD’ residues of the neighboring subunit (Arg367’) where helix α3 (that precedes the important functional element loop1 ^2^) takes part to the hinge with helix α1 of the same subunit (by the saline bond Arg27-Glu61) and is contacted on the back part by the CTD of the facing monomer (**Fig. S10C**). These different conformations in the NTD region could mirror different conformations among subunits in the entire protein. AADC shows that the entire loop3 (containing the CL) is completely solvent exposed in both subunits with Tyr332’ rolled out and the region including residues 348’-358’ (at the end of loop3’) approaching the facing CTD (**Fig. S11A-C**). Faint differences are visible between the two subunits with one CTD favoring loop3 of the facing subunit to approach sheet β10. Instead, in holoAADC, some of loop3’ residues flanking the CL (Asp345’, Arg347’, Gln350’, Pro352’, Leu353’), are buried and in contact with part of the opposite active site (Fig. **S11D-F**). Interestingly, CL displays a marked fluctuation exploring two conformations. In monomer 1, the CL faces the opposite active site (CL-in) at a contact distance with respect to sheet β10 of CTD, while the catalytic Tyr332’ bends towards PLP of the opposite subunit (**Fig. S11E**). On the *re*-face of the cofactor, His192 forms a π-stacking interaction with the pyridine ring while Phe80 moves to a T-shaped conformation on the *si*-face and Phe309 tilts of 90° (**Fig. S12A,B)**. His192 sandwiches PLP in a state able to function as electron sink, in a conformation similar to that observed in the solved crystal structure of the internal and external aldimine (see above). The CL of monomer 2 is twisted outside the active site (CL-out) being exposed to the solvent and, notably, the facing sheet β10 of the CTD is locally disordered (**Fig. S11F**). This conformation is reflected at the active site with His192 assuming a catalytically disfavored T-shaped conformation and Phe80 forming a π-stacking interaction with the PLP pyridine (**Fig. S12C,D**). The central cavity that interconnects the active sites shows an asymmetric distribution of volume and differences between apo and holoAADC (**Fig. S12E-G**). Interestingly, residues surrounding this cavity belong to the CTD (His439), to loop3 (Thr331, Tyr332, Leu353, Arg358), to the PLP binding cleft of the LD (Glu150, Ser147, Ser193 and His192), and many of them have been seen above to play important structural and functional roles.

**Coarse-Grained (CG) Molecular Dynamics (MD) simulations**

We calculated the 2D average distance maps between several structural elements of apo and holo-like AADC: i) NTD of chain A and the related CTD of the same chain, and the opposite ones, ii) NTD of chain A and loop2’ of chain B and the opposite ones, iii) NTD of chain A and loop3’ of chain B and the opposite ones, iv) CTD of each chain with loop3’ of the opposite one. The distances of the residues between NTD (residues 1-64, comprising helix α1-loop-helix α2-loop-helix α3) and CTD (residues 417-440, belonging to helix α16 and sheet β10) are longer for apo and approach in holo-like species. In addition, all chains in the two species present a marked asymmetry. Notably, the segment comprising the peptide bond Arg27-Gln28 is outdistanced to helix α16 and sheet β10 in the apo structure, while it seems to be closer, even if asymmetrically for the two subunits, in the holo-like species (**Fig. S13C).** These differences between apo and holo-like species are in agreement to their dissimilar behavior in proteolysis at Arg27. Regarding distances of each NTD with respect to loop2’ and loop3’ of the opposite subunit, despite the displayed asymmetry, it is evident how the Arg27 region is far from these loop elements in the apo enzyme, while the same NTD region is nearer to loop2’ (but not loop3’) of the opposite subunit in the holo-like species **(Fig. S13B)**. The CTD is more detached from loop3’ in the apo than in the holo-like species, suggesting a more elongated conformation for the apo species **(Fig. S13B).** Interestingly, all CGMD AADC model structures show asymmetric behavior, in line with the all-atom MD simulations.

**Supplementary Materials and Methods**

**Site-directed mutagenesis**

AADC variants were obtained by mutating the template DNA on the pAADChis vector as previously described ^11^. Each mutagenesis reaction has been performed using the Quick-Change II kit (Agilent technologies) using the appropriate oligonucleotides (the changed nucleotides are underlined):

R27A: 5’-CATTGAGGGAGCCCAGGTCTACCCTG-3’;

R27Q: 5’-CATTGAGGGACAGCAGGTCTACCCTG-3’;

E61A: 5’- CGACGTTGCGAAGATAATCATGCCTGGGG-3’;

K334Q: 5’-CCCCACTTACCTGCAGCACAGCCATCAGGA-3’;

E150A: 5’-GAAGTGCCAGTGCAGCCACCCTGGTG-3’;

E150Q: 5’-GAAGTGCCAGTCAAGCCACCCTGGTG-3’).

All mutations were confirmed by DNA sequence analysis of the whole ORF.

**Recombinant protein production and purification.**

*E. coli* BL21(DE3) cells were transformed by heat shock at 42 °C with the desired construct and grown in 4.5 L of Luria–Bertani (LB) broth in presence of 100 mg/mL ampicillin. The cultures were grown at 37 °C to optical density 600 of 0.4–0.6 and induced with 0.1 mM IPTG. The cultures were maintained at 30 °C for additional 15 h. Cells were harvested by centrifugation and resuspended in 20 mM sodium phosphate buffer pH 7.4, containing 0.5 M NaCl, 20 mM imidazole, 50 μM PLP, 0.5 mM PMSF and protease inhibitor cocktail. Cell lysis was performed with the addition of 0.2 mg/mL lysozyme for 1 h at 25 °C. After a freeze thaw, leupeptin and pepstatin (both at 1 μg/mL concentration) were added and the suspension was centrifuged at 16,000 g for 30 min. The crude cellular lysate was loaded on a HisPrep FF 16/10 column (GE Healthcare) pre-equilibrated with 20 mM sodium phosphate buffer pH 7.4, containing 0.5 M NaCl and 20 mM imidazole. AADC was then eluted with a linear gradient (0–100% in 100 mL) of the same buffer containing 500 mM imidazole. AADC fraction was concentrated and imidazole and unbound coenzyme were removed by extensive washing with 100 mM potassium phosphate buffer pH 7.4, using Amicon Ultra 15 concentrators (Millipore). Protein concentration was determined using an ε_M_ of 1.42·10^5^ M^−1^ cm^−1^ at 280 nm. PLP/AADC ratio was assessed by releasing the PLP in 0.1 M NaOH using ε_M_ of 6600 M^−1^ cm^−1^ at 388 nm.

**Coenzyme binding affinity measurements**

The apparent equilibrium dissociation constant for PLP, K_D(PLP)_, was determined as follow. Apo proteins were obtained by incubating 5 μM holoenzymes with 15 mM hydroxylamine in 0.5 M potassium phosphate buffer pH 6.8 at 25 °C for 15 h. The protein solutions were loaded on a Desalting 26/10 column (GE Healthcare) pre-equilibrated with 0.5 M potassium phosphate buffer pH 6.8, eluted at 1 mL/min and then concentrated on Amicon Ultra 15 concentrators (Millipore). 100 nM apo AADC samples were then incubated in the presence of increasing PLP concentrations (from 0.005 to 20 μM) for 15 h at 25 °C (in the dark) in 100 mM potassium phosphate buffer pH 7.4. Fluorescence spectra were recorded on a Jasco FP-8500 fluorimeter by exciting the protein samples at 280 nm and the progressive quenching of the intrinsic emission was then fitted to the following equation:

$$Y=Y_{max}\frac{\left[ E \right]t+\left[ PLP \right]t+KD(PLP)-\sqrt{\left( \left[ E \right]t+\left[ PLP \right]t+KD(PLP) \right)^{2}-4[E]t[PLP]t}}{2[E]t}$$

where [E]t and [PLP]t represent the total AADC and PLP concentrations, respectively, Y refers to the intrinsic quenching changes at each PLP concentration, and $Y_{max}$refers to the plateau relative to a protein sample with all molecules complexed with the coenzyme. Curves fitting was performed using Prism, 8.4.0, (GraphPad ®). Curves fitting was performed using Prism, 8.4.0 (GraphPad), on three independent experiments. Values obtained are the mean ± standard error of the mean (SEM)).

**Kinetic parameters for L-Dopa**

The kinetic parameters for the decarboxylation of L-Dopa of AADC variants R27A, R27Q, E61A, K334Q, were determined by HPLC as reported in ^17^. HPLC assay method was used also to assess the activity of previously reported pathogenic variants L38P ^18^, A110E ^11^  and L353P ^11^, whose activity was below the sensitivity of the previous assay method. Each variant was pre-incubated with a concentration of PLP 10 times higher the evaluated *K*_D(PLP)_. The kinetic parameters were calculated within an appropriate L-Dopa concentration range (according to the Km) with the reaction time and enzyme concentration set in order to detect a linear dopamine formation. The final reaction volume was 225 μL in 100 mM potassium phosphate buffer pH 7.4. The mixtures were then quenched with 25 μL of a 100 % TCA solution. Proteins were precipitated in ice and removed by centrifugation. Supernatants were analyzed by HPLC using a Gemini C18 column (150 Å, 4.6 mm, Phenomenex, CA, USA) on a Jasco PU-2080 Plus HPLC system equipped with a UV-1570 detector set at 295 nm. Samples were eluted in 100 mM potassium phosphate, pH 2.35, at a flow rate of 1 mL/min. Standard curves of dopamine peak area were prepared with commercially available dopamine. The kinetic parameters of each AADC variant were carried out in triplicate and determined by fitting the data to the Michaelis-Menten equation using Prism, 8.4.0, (GraphPad). Data are reported as mean ± SEM.

**Spectroscopic measurements**

All spectral measurements were acquired in 100 mM potassium phosphate, pH 7.4, with 100 μM of exogenous PLP at 25 °C. CD measurements were recorded with a Jasco J-715 spectropolarimeter equipped with a Grant LTC2 refrigerated circulator.

Near UV-visible spectra were recorded at a scan speed of 50 nm/min, band width 1 nm, response 4 sec, data pitch 1 nm, cell length 1 cm, and at a protein concentration of 0.5 mg/mL. All AADC species were repeated in triplicate with each spectrum resulted from the average of 3 accumulations.

Thermal denaturation was performed by monitoring the dichroic signal of 0.4 mg/mL protein sample at 222 nm. The instrument was set with a band width of 1 nm, response 4 sec, data pitch 0.2 °C, cell length 0.1 cm and a temperature gradient was applied in the range between 25-90 °C with a temperature slope of 1 °C/min.

**Limited proteolysis experiments with trypsin**

Trypsin digestion of 0.54 mg/mL AADC was performed in 100 mM potassium phosphate, pH 7.4 at 25 °C at the final E/S ratio of 1:100 (w/w). 100 μM PLP was added to the holo species. Aliquots were withdrawn at different times and compared with AADC sample in the absence of trypsin. At each time point, the reactants were boiled in SDS sample buffer to stop the proteolytic activity. The digestion products were separated by SDS-PAGE, and gels were stained with Coomassie. The gels were scanned, and the peak profiles were obtained for the bands using IMAGE J software (National Institutes of Health). All species were calculated as percentage of the initial undigested band and were plotted against time. Monomer disappearance was fitted to a simple exponential to obtain the half-life and the initial rate (V_0_) of the full length AADC cleavage. Digestions were performed as triplicate and the calculated rates are reported as mean ± SEM. Reconstitution experiments of cleaved AADC species were performed by adding trypsin inhibitor at the ratio 1:2 (w/w) ratio after 1 hour of digestion. Unpaired t-test statistical analysis was conducted using Prism, 8.4.0, (GraphPad). ns *p* > 0.05, * *p* ≤ 0.05, ** *p* ≤ 0.01, **** *p* ≤ 0.0001.

**Identification of the tryptic sites by LC-MS**

A solution containing 0.54 mg/mL of AADC has been digested with trypsin at 1:100 (w/w) final E/S ratio in 100 mM potassium phosphate, pH 7.4 at 25 °C, with the addition of 100 μM PLP for the holo enzymes. Digestion was quenched after 30 min by adding specific trypsin inhibitor at the ratio 1:2 (w/w) ratio. Reaction mixture was then desalted and analyzed by RP-HPLC (Agilent, mod. 1200, Santa Clara, Ca, USA) with a Jupiter C4 column (4.6 mm x 250 mm, 5 μm; Phenomenex, CA, USA). Peptides peaks were eluted with a gradient of water and acetonitrile, containing 0.1 % of trifluoroacetic acid: from 5 to 38 % in 5 min and from 38 to 43 % in 15 minutes at 226 nm. Peptide identity was assessed with a Xevo. G2-XS ESI-Q-TOF mass spectrometer (Waters Corporation, Milford, Massachusetts, USA) in positive mode. The capillary potential was set at 1.5 kV. The source

temperature was at 100 °C. Mass values were determined at a resolution >35.000 and an accuracy <5 ppm. Mass spectra were analyzed by the Mass-Lynx 4.1 software (Waters).

Table S1. Data collection and refinement statistics.

| **RBP4 Data set** | **Holo human AADC** | **Holo human AADC saturated with DME** |
| --- | --- | --- |
| Space group | P6_1_22 | P6_1_22 |
| Crystal form | 3 | 8 |
| a (Å) | 107.17 | 107.64 |
| b (Å) | 107.17 | 107.64 |
| c (Å) | 218.98 | 218.24 |
| α | 90.0 | 90.0 |
| β | 90.0 | 90.0 |
| γ | 120.0 | 120.0 |
| Molecules in the asymmetric unit | 1 | 1 |
| Resolution range (Å) | 85.45 - 1.90 | 48.27 - 2.40 |
| Observed reflections | 341,933 | 396,403 |
| Independent reflections | 59,247 | 28,904 |
| Multiplicity | 5.8 (6.0) | 13.7 (14.2) |
| Rmerge (%) | 10.6 (47.4) | 10.5 (50.7) |
| <I/σ(I)> | 10.1 (3.0) | 17.6 (5.1) |
| Completeness (%) | 99.9 (100.0) | 97.2 (98.3) |
| Reflections in refinement | 59,121 | 28,886 |
| Rcryst (%) | 19.05 | 21.27 |
| Rfree (%) (test set 5%) | 21.54 | 22.66 |
| Protein atoms | 3,667 | 3,790 |
| Ligand atoms | 28 | 43 |
| Water molecules | 250 | 111 |
| R.m.s.d. on bond lengths (Å) | 0.002 | 0.002 |
| R.m.s.d. on bond angles (Å) | 0.527 | 0.505 |
| Planar groups (Å) | 0.004 | 0.004 |
| Chiral volume dev. (Å^3^) | 0.041 | 0.040 |
| Average B factor (Å^2^) | 24.59 | 42.67 |
| Protein atoms | 24.28 | 42.75 |
| Ligand atoms | 29.38 | 46.96 |
| Solvent atoms | 28.57 | 38.37 |
| Synchrotron beamline  Date of data collection | Elettra – XRD2 28/01/2020 | ESRF – ID23-2 28/11/2021 |
| PDB code | 8OR9 | 8ORA |

* The values in parentheses refer to the highest resolution shells.

^a^Rmerge = Σ*h*Σ*i* | I*ih* – <I*h*> / Σ*h*Σ*i* <I*h*> where <I*h*> is the mean intensity of the *i* observations of reflection *h*.

^b^Rcryst = Σ | |Fobs| - |Fcalc| | / Σ |Fobs|, where |Fobs| and |Fcalc| are the observed and calculated structure factor amplitudes, respectively. Summation includes all reflections used in the refinement.

^c^Rfree = Σ | |Fobs| - |Fcalc| | / Σ |Fobs|, evaluated for a randomly chosen subset of 5% of the diffraction data not included in the refinement.

^d^Root mean square deviation from ideal values.

**Table S2.** Parameters estimated from SAXS data of apo, holo and DME-bound AADC.

| **Sample** | ***q_max_***  **(Å^-1^)** | **Data resolution (Å)** | ***R_g_* reciprocal space**  **(Å)** | ***R_g_* real space**  **(Å)** | ***D_max_***  **(Å)** | **Porod-Debye**  **Volume**  **(Å^3^)** | **Porod**  **exponent** | **MW**  **(kDa)** |
| --- | --- | --- | --- | --- | --- | --- | --- | --- |
| apo | 0.18 | 33.5 | 34 | 37 | 158 | 237728 | 2.9 | 121 |
| holo | 0.27 | 26.2 | 32 | 34 | 149 | 194873 | 3.1 | 108 |
| DME-bound | 0.16 | 26.1 | 30 | 31 | 145 | 179537 | 3.1 | 98 |

Maximum momentum transfer (*q_max_*) and data resolution estimated for the dataset, radius of gyration (*R_g_*) from Guinier analysis (reciprocal space) and pair distribution function determination (real space), maximum inter-particle distance (*D_max_*), molecular weight estimated by using the Porod approach (MW) are shown.

**Table S3.** Mass determination of proteolytic fragments of his-tagged holo and apoAADC obtained by ESI-MS analysis.

| **Holo**  **MW (Da)** | **Apo**  **MW (Da)** | **Theoretical**  **MW (Da)** | **Identified**  **species (amino acids)** |
| --- | --- | --- | --- |
| 54718.08 | 54720.03 | 54717.13 | **1-486** |
| 36624.05 | 36625.51 | 36624.11 | **1-334** |
| 18112.01 | 18112.01 | 18112.04 | **335-486** |
| -- | 3208.13 | 3209.62 | **1-27** |
| -- | 33431.55 | 33432.50 | **28-334** |
| -- | 51526.51 | 51525.53 | **28-486** |
| 17159.52 | 17159.02 | 17159.08 | **335-479** |

**Table S4:** Kinetic parameters for L-Dopa and equilibrium binding dissociation constant for PLP (*K*_D(PLP)_) for WT AADC and R27A, R27Q, E61A, E150A, E150Q, K334Q variants.

| **Enzyme** | ***k*_cat_**  **(s^-1^)** | **K_m_**  **(mM)** | ***k*_cat_/K_m_**  **(s^-1^mM^-1^)** | **K_D(PLP)_**  **(nM)** | **holo Tm**  **222 nm (°C)** | **apo Tm**  **222 nm (°C)** |
| --- | --- | --- | --- | --- | --- | --- |
| **WT** | 5.5 ± 0.1^a^ | 0.016 ± 0.001^a^ | 343 ± 22 ^a^ | 101 ± 10 ^a^ | 68.16 ± 0.19 | 62.86 ± 0.27 |
| **R27A** | 0.37 ± 0.03 | 0.016 ± 0.001 | 23 ± 2 | 438 ± 86 | 64.58 ± 0.17 | 61.47 ± 0.06 |
| **R27Q** | 2.40 ± 0.02 | 0.017 ± 0.001 | 141 ± 8 | 493 ± 62 | 65.57 ± 0.17 | 62.26 ± 0.22 |
| **E61A** | 0.62 ± 0.05 | 0.029 ± 0.001 | 21 ± 2 | 307 ± 44 | 66.27 ± 0.43 | 61.68 ± 0.29 |
| **E150A** | 2.9 ± 0.2 | 0.79 ± 0.05 | 3.7 ± 0.3 | 215 ± 21 | n.d. | n.d. |
| **E150Q** | 2.4 ± 0.3 | 1.23 ± 0.6 | 2.1 ± 0.6 | 372 ± 42 | n.d. | n.d. |
| **K334Q** | 4.26 ± 0.08 | 0.018 ± 0.002 | 237 ± 27 | 104 ± 21 | 68.07 ± 0.29 | 63.08 ± 0.25 |

All measurements were performed in 0.1 M potassium phosphate buffer, pH 7.5 at 25°C. Data are reported as mean ± SEM and are obtained from three independent experiments.

^a^taken from ^17^, n.d., not determined

**Table S5:** Initial rate and half-life of 54.7 kDa apo and holo AADC species.

|  | **V_0_ (min^-1^)** | | **Half-life (min)** | |
| --- | --- | --- | --- | --- |
| **Enzyme** | **holoAADC** | **apoAADC** | **holoAADC** | **apoAADC** |
| **WT** | 5.6 ± 0.7 | 8.3 ± 0.2 | 12.8 ± 1.7 | 8.3 ± 0.2 |
| **R27A** | 11.4 ± 0.9 | 10.8 ± 0.3 | 6.2 ± 0.5 | 6.4 ± 0.2 |
| **R27Q** | 7.2 ± 0.6 | 7.9 ± 0.8 | 9.7 ± 0.8 | 9.9 ± 0.5 |
| **E61A** | 17.1 ± 1.4 | 18.9 ± 0.5 | 4.2 ± 0.6 | 3.8 ± 0.1 |
| **K334Q** | 0.0 | 3.7 ± 0.2 | ***--*** | 19.2 ± 1.0 |

Values were calculated from SDS-PAGE of limited proteolysis carried out with WT and variant AADC species. Results are reported as the mean ± SEM of three independent experiments.

**Table S6:** Fold-change in the catalytic parameters *k_cat_*, K_m_, *k_cat_*/K_m_ of AADC deficiency pathogenic variants with respect to WT AADC.

| **Variant** | **Fold change in *k*_cat_** | **Fold change in Km** | **Fold change in *k*_cat_/Km** | **reference** |
| --- | --- | --- | --- | --- |
| **L38P** | 9482±676 | 15±2 | 144019±20534 | ^18^ and this work |
| **P47H** | 4.5±0.2 | 5.5±0.5 | 24±2 | ^18^ |
| **V60A** | 8.2±0.2 | 1.5±0.1 | 11.9±0.8 | ^11^ |
| **T69M** | 2.2±0.1 | 4.2±0.6 | 9±1 | ^19^ |
| **H70Y** | 13±2. | 2.4±0.4 | 31±7 | ^18^ |
| **H72Y** | 76±8 | 12±2 | 954±186 | ^18^ |
| **F77L** | 22±4 | 4.6±0.5 | 101±22 | ^11^ |
| **Y79C** | 23.8±0.8 | 36±3 | 859±86 | ^18^ |
| **P81L** | 15.2±0.4 | 1.7±0.2 | 26±3 | ^18^ |
| **A91V** | 916±78 | 1.4±0.1 | 1249±136 | ^20^ |
| **G96R** | 2.0±0.4 | 16.5±0.4 | 34±6 | ^11^ |
| **C100S** | 1.1±0.1 | 3.8±0.9 | 4±1 | ^21^ |
| **G102S** | 6.3±0.5 | 10.9±0.9 | 69±8 | ^22^ |
| **A110E** | 17741±618 | 5.6±0.6 | 99798±11688 | ^11^ and this work |
| **G123R** | 2.3±0.1 | 6.7±0.6 | 16±2 | ^18^ |
| **S147R** | 844±22 | 1.1±0.1 | 921±81 | ^19^ |
| **R160W** | 6.8±0.4 | 18±2 | 120±17 | ^23^ |
| **P210L** | 1.70±0.08 | 1.1±0.2 | 1.9±0.3 | ^11^ |
| **S250F** | 3.6±0.2 | 2.0±0.4 | 7±1 | ^18^ |
| **F251S** | 1.9±0.1 | 0.8±0.1 | 1.5±0.2 | ^11^ |
| **W267R** | 3.6±0.1 | 1.7±0.1 | 6.2±0.4 | ^11^ |
| **A275T** | 5.4±0.2 | 3.2±0.3 | 17±2 | ^22^ |
| **E283A** | 1.2±0.1 | 2.6±0.5 | 3.1±0.6 | ^11^ |
| **R285W** | 3.7±0.1 | 1.5±0.3 | 5±1 | ^18^ |
| **F309L** | 16±1 | 44±5 | 706±100 | ^22^ |
| **P330L** | 189±4 | 3.6±0.1 | 673±28 | ^17^ |
| **R347Q** | 87±5 | 4.5±0.7 | 389±68 | ^18^ |
| **R347G** | 475±30 | 6±1 | 2764±590 | ^8^ |
| **L353P** | 8871±866 | 16±2 | 144153±21866 | ^11^ and this work |
| **R358H** | 253±9 | 44±4 | 11286±1016 | ^23^ |
| **M362T** | 1.7±0.1 | 1.6±0.5 | 2.7±0.9 | ^19^ |
| **L408I** | 9.7±0.8 | 16±2 | 158±26 | ^18^ |
| **C410G** | 1.7±0.1 | 2.5±0.4 | 4.2±0.7 | ^20^ |
| **R412W** | 5.2±0.2 | 2.5±0.3 | 13±2 | ^18^ |
| **R447H** | 20±1 | 7±1. | 144±25 | ^18^ |
| **R453C** | 11±1 | 3.6±0.5 | 41±8 | ^11^ |
| **R462P** | 19.0±0.5 | 2.3±0.3 | 43±5 | ^18^ |

Raw data can be found in the cited papers, for catalytic parameters of L38P, A110E and L353P see legend of Figure 6 of the main text. Residues are listed on the basis of the amino acid sequence and differently colored by domain: cyan, NTD; green, LD; blue, CTD.

**Fig. S1. Crystal structure of human native holoAADC.** A) The PLP-Lys303 internal aldimine is represented as spheres and the unstructured regions loop1, loop2 and loop3 are colored yellow, red and blue, respectively. The number of residues belonging to the loops in this structure is slightly different from ^2^, not unexpected since this is a holo structure where PLP presence can contribute to stabilization of structural elements. The highly flexible stretch of residues belonging to the CL are missing in the electron density map. Remarkably, loop1 is sandwiched between NTD and CTD of the same subunit while loop2 and loop3 extend towards the active site of the second subunit. B) Cartoon representation of the structure of homodimeric holoAADC. NTD is cyan, LD is green and CTD is blue for one of the two subunits. One of the two active sites is circled and colored yellow. The dimeric structure has been generated by applying the symmetry operator to the crystallographic subunit and visualized by using PyMOL.

**Fig. S2.** **Crystal structure of the internal (white) and external (yellow) aldimine intermediates at the active site of holoAADC.** A) PLP-Lys303 Schiff-base electron density contoured at 1.0σ together with main polar interactions of residues involved in PLP binding. Crystallographic water molecules are rendered as red spheres and named with the alphabetic letters. The prime denotes residue(s) from the neighboring subunit. B) Active site global architecture including loop 1, loop2 and loop3 residues visible in the electron density map. The PLP pocket and the entrance gorge of the active site are visualized as a surface. C) Electron density map of the internal aldimine and the visible part of loop3 whose residues 342-357 are reported in the bottom part. D) Focus on the inter-subunit interactions between residues of the active site and residues of loop3’ protruding inside it in native holoAADC. E) PLP-DME external aldimine and Lys303 electron density contoured at 1.0σ. Only residues making polar contacts are shown. F) Active site global architecture including loop 1, loop2 and loop3 residues visible in the electron density map in the external aldimine conformation. G) Electron density map of Lys303, PLP-DME and complete CL. Residues 327-341 are reported in the bottom part. H) Intramolecular and intermolecular interactions established by CL residues protruding inside the opposite active site in the external aldimine conformation. I) Electrostatic surface potential of the AADC active site in its internal and external aldimine conformations generated with APBS (Adaptive Poisson-Boltzmann Solver) and represented using PyMOL, calculated at pH 7.4, colored between +5 (blue) and -5 (red) kT/e^-^.

**Fig S3. Comparison between human α-decarboxylases: AADC, HDC and GAD67 CL crystal conformation**. A) Cartoon representation of superposed dimeric AADC (yellow), HDC (green) and GAD-67 (magenta). B) Closed conformation of the CL with the catalytic tyrosine residue (position 332 in AADC, 334 in HDC and 434 in GAD-67) in close proximity to the conserved histidine residue (position 192 in AADC, 194 in HDC and 291 in GAD-67) and the PLP-ligand complex (L-Dopa methylester and histidine methylester in AADC and HDC, respectively) or GABA in GAD-67. C) Involvement of NTD and CTD in CL positioning of AADC, HDC and GAD-67, colors are the same as above.

**Fig S4. Features of the AADC dimer interface**. A) Cartoon representation of the X-ray structure of one AADC subunit with the interface residues represented as blue sticks. Both PLP-DME complexes are shown as spheres and colored by element. B) Surface representation of dimeric native AADC sliced on a plane orthogonal to the 2-fold axes. The active site gorges and the connecting central water chamber are contoured with a drawn black line. PLP and residues are represented as ball and sticks, the water molecules as red spheres and the polar interactions as dashed lines. The prime denotes elements belonging to subunit 2. Loop1 and helix-α4 are shown as cartoon. C) Surface representation of dimeric DME-bound AADC. External aldimine PLP-DME and closed CL are also represented. D) AADC molecular surface colored by conservation score calculated with the ConSurf web server (see Methods section) with a gradient from 1 (white, variable) to 9 (red, conserved). The histogram presents the conservation score value present in residues not belonging (magenta) or belonging (yellow) to the interface F) Ribbon representation of one AADC subunit colored by the crystallographic thermal B-factor. PLP-DME complex is shown as spheres. Ribbon thickness is proportional to atomic B-factor.


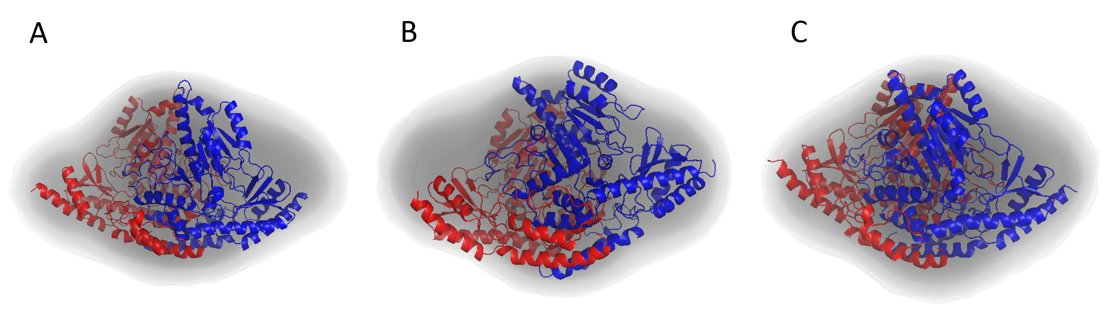


**Fig. S5. Validation of the SAXS-derived AADC structural models.** Structural models refined by flexible-fitting molecular dynamics (cartoon representation, with the two chains in blue and red) superposed to the molecular envelope calculated from SAXS data by using the DENSS approach (transparent surface) for apo A), holo B) and DME-bound C) AADC.

**Fig. S6. Comparative analysis of the geometrical features of the AADC structural models.** A) Scores plot of the first two principal components PC1 and PC2. The percentage of data variance explained is reported on the axes. 85% confidence levels ellipses show the results of a hierarchical clustering. B) Scores of PC1 and PC2 plotted separately. C) Loadings of PC1 and PC2, showing the contribution of each geometrical descriptor to the principal component. D) Schemes showing the definition of the geometrical descriptors, where the residues Glu173 and Leu470 of the two chains are shown in green and yellow, respectively. See the text for the description.

**Fig S7. Limited proteolysis of apo, holo and DME-bound AADC.** A) Recombinant purified AADC has been incubated with trypsin at E:S ratio of 1:100 (w:w) in 0.1 M potassium phosphate buffer pH 7.5 at 25ºC. AADC was pre-incubated with 100 μM PLP for the holo species or 5 mM DME for the DME-bound species. At different times (minutes 1, 2, 5, 10, 20, 40, 60) aliquots of the reaction mixture were withdrawn, boiled in SDS sample buffer and loaded onto SDS-PAGE. Time zero is referred to the AADC sample not incubated with trypsin. Molecular weight markers (MW) are on the left. B) Time course of full length AADC species disappearance evaluated as percentage of initial undigested 54.7 kDa monomer. The experimental points are shown in circles and represent the mean of three independent replicates, while the solid lines indicate the exponential fitting curve of the mean values. Error bars indicate SEM and are frequently smaller than the symbols. C) Proposed pattern of proteolysis for apo and holoAADC in accordance to the identified tryptic sites. D) Superposition of the crystal structures of apo (PDB: 3RBL) and holoAADC (PDB: 8OR9), HDC (PDB: 4E1O), GAD-65 (PDB: 2OKK), GAD-67 (PDB: 2OKJ) and CSAD (PDB: 2JIS) in the NTD-CTD region of the NTD tryptic site. The alignment of the sequences of the homologous α-decarboxylases (obtained by Clustal Omega, https://www.ebi.ac.uk/Tools/msa/clustalo/) in this region is reported below.

**Fig S8. Near UV-visible dichroic spectra and limited proteolysis experiments of R27Q, R27A, E61A, K334Q AADC variants.** A) Near UV-visible dichroic spectra of holo and apoAADC WT and variant species. Each spectrum is the average of three accumulations of three different samples recorded at 5 μM protein concentration. B) Limited proteolysis of R27Q, R27A, E61A, K334Q AADC variants. Time zero is referred to the AADC sample in the absence of trypsin. At the indicated times (1, 2, 5, 10, 20, 40, 60 minutes), aliquots were withdrawn, denatured by boiling, treated with SDS sample buffer and loaded onto SDS-PAGE. At the bottom of each SDS-PAGE, the rate of disappearance of the full length (54.7 kDa) apo (blue) and holo (black) species is plotted versus time. Symbols represent the mean of three independent experiments, while the solid lines indicate the exponential fitting curve. Error bars indicate SEM and are frequently smaller than the symbols. C) Comparison of the initial rates of disappearance of full length (54.7 kDa) AADC among WT and variants. Results derive from three independent experiments (white open circles) and bars indicate mean ± SEM; ns *p* > 0.05, * *p* ≤ 0.05, ** *p* ≤ 0.01, **** *p* ≤ 0.0001. D) Limited proteolysis of apoK334Q carried out up to 4 hours. Filled blue circle represents full-length 54.7 kDa apoK334Q, open blue triangle represents the 51.5 kDa product. Symbols represent the mean of three independent experiments. Error bars indicate SEM and are frequently smaller than the symbols. E) Time course accumulation of the 51.5 kDa AADC band for apoWT, apoE61A, holoE61A and apoK334Q AADC. Symbols represent the mean of three independent experiments. Error bars indicate SEM and are frequently smaller than the symbols. All experiments were performed in triplicate in potassium phosphate buffer 0.1 M pH 7.4 at 25 ºC. Holo species were pre-incubated in the presence of 100 μM PLP.

**Fig S9. Near UV-Vis dichroic spectra and residual activity of apo and holo WT, K334Q and R27Q AADC in the absence or presence of PLP and untreated or treated with trypsin.** Apo and holoWT, K334Q and R27Q were incubated with trypsin at E:S ratio of 1:100 (w/w) in potassium phosphate buffer 0.1 M pH 7.4 at 25 ºC (in the presence of 100 μM PLP for the holo forms) and the digestion was then stopped after 60 minutes with the addition of trypsin specific inhibitor at the ratio 1:2 (w/w). Digested mixtures (+ trypsin) were then compared to the undigested ones. A) Near UV-visible dichroic spectra of apo and holoWT, K334Q and R27Q AADC native or treated with trypsin in the absence or presence of PLP. Each spectrum is the average of three accumulations for three different samples recorded at 5 μM protein concentration. B) Residual activity (%) of digested and undigested WT AADC and variants measured in the presence of saturating concentration of L-Dopa. Results derive from three independent experiments (white symbols) and bars indicate mean ±SEM.

**Fig. S10. All-atom MD simulations models focused in the region of the Arg27-Glu61 salt bridge.** A) Solvent accessible surface area of Arg27 during all-atom MD simulations. B) Interdistance between atoms C_Z_ of Arg27 and C_D_ of Glu61 throughout the all-atom MD simulation time for the two polypeptide chains of both apo and holoAADC. C) Intra- and inter-subunit network of interactions in the region involving NTD-CTD contacts in holo and apoAADC all-atoms MD models, NTD (magenta), CTD (orange).

**Fig. S11. All-atom MD simulations of dimeric apo and holoAADC.** A) Superposition of the first (yellow) and last (red) MD frames of apoAADC. B) zoom of the first and C) second active site with focus on the loop3 and CTD relative positions. D) Superposition of the first (blue) and last (cyan) MD frames of holo AADC. B) zoom of the first (CL-in) and D) second (CL-out) active site with focus on the loop3 and CTD relative positions.


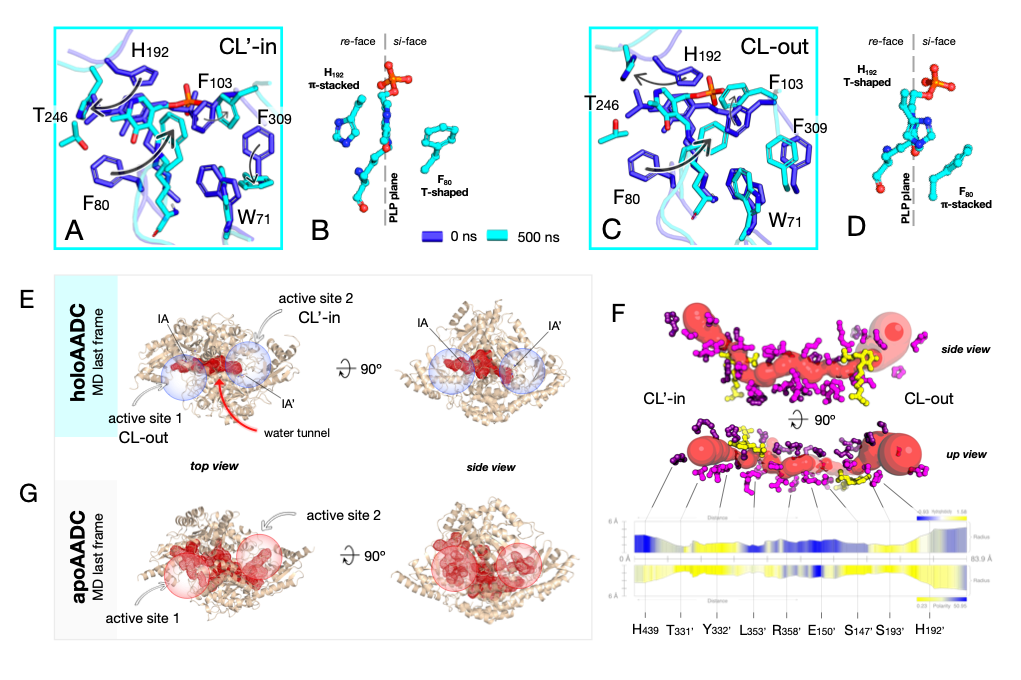


**Fig. S12. All-atom MD simulations at the two active sites of holoAADC and connection water room among the active sites in both apo and holoAADC.** A) and B) different positions of relevant residues at the active site 1 (CL-in) with geometric orientation with respect to the pyridine plane, C) and D) different positions of relevant residues at the active site 2 (CL-out) with geometric orientation with respect to the pyridine plane (last frame of all-atom MD simulations). E) holo and F) apoAADC MD frame at 500 ns revealing the asymmetry of the active site communication room represented as volume of water molecules. Differences are not only in terms of the occupied volume (1500 Å^3^ for holo and 5600 Å^3^ for apo) but also in asymmetry. G) Computation by MOLEonline (https://mole.upol.cz) from the last MD frame of the asymmetric amino acid composition of the holoAADC tunnel whose central part is mainly hydrophilic while both active sites are strongly apolar. Residues involved in the tunnel are labeled below, the prime denotes a residue belonging to the neighboring subunit.

**Fig. S13. RMSD and 2D maps of CGMD simulations of apo and holo-like AADC.** A) RMSD of the backbone of each AADC chain obtained for each of the three apo and holo-like CGMD replicas. Simulations were performed for a total time of 20 μs. B) AADC molecular shape represented as surface and obtained by clustering all simulation frames after the first 10 μs of equilibration. C) 2D distance map representation of the apo and holo-likeAADC. Both intrachain and interchain matrices are reported.

**Movie S1 (separate file).** Trajectory of a 500 ns simulation of holo and apoAADC in all-atoms MD simulations. The two dimeric structures are displaced in a 2 grids panel, holoAADC on the left and apoAADC on the right. ChainA and chainB of each protein species are rendered with different colors. The movie shows the first rearrangement of the initial crystallographic structures and focus on the NTD-CTD edge at about 450 ns of simulations and residues discussed in the text are shown. For visualization clarity, water molecules and ions are not shown. Image smoothing was performed with a window sized of 3 frames, which may have produced slight distortion of certain structures.

**Movie S2 (separate file).** A simplified view of the two asymmetric holoAADC active sites showing the closed and open CLs and some active site elements (described in the text) are here displayed during the simulation time. Water molecules, ions and the other part of the protein are not shown. Image smoothing was performed with a window sized of 3 frames, which may have produced slight distortion of certain structures.

**SI References**

1. Burkhard P, Dominici P, Borri-Voltattorni C, Jansonius JN, Malashkevich VN. Structural insight into Parkinson's disease treatment from drug-inhibited DOPA decarboxylase. *Nat Struct Biol.* 2001;8(11):963-967.

2. Giardina G, Montioli R, Gianni S, et al. Open conformation of human DOPA decarboxylase reveals the mechanism of PLP addition to Group II decarboxylases. *Proc Natl Acad Sci U S A.* 2011;108(51):20514-20519.

3. Paiardini A, Giardina G, Rossignoli G, Voltattorni CB, Bertoldi M. New Insights Emerging from Recent Investigations on Human Group II Pyridoxal 5'-Phosphate Decarboxylases. *Curr Med Chem.* 2017;24(3):226-244.

4. Bertoldi M, Gonsalvi M, Contestabile R, Voltattorni CB. Mutation of tyrosine 332 to phenylalanine converts dopa decarboxylase into a decarboxylation-dependent oxidative deaminase. *J Biol Chem.* 2002;277(39):36357-36362.

5. Grishin NV, Phillips MA, Goldsmith EJ. Modeling of the spatial structure of eukaryotic ornithine decarboxylases. *Protein Sci.* 1995;4(7):1291-1304.

6. Jansonius JN. Structure, evolution and action of vitamin B6-dependent enzymes. *Curr Opin Struct Biol.* 1998;8(6):759-769.

7. Bertoldi M, Castellani S, Bori Voltattorni C. Mutation of residues in the coenzyme binding pocket of Dopa decarboxylase. Effects on catalytic properties. *Eur J Biochem.* 2001;268(10):2975-2981.

8. Montioli R, Paiardini A, Kurian MA, et al. The novel R347g pathogenic mutation of aromatic amino acid decarboxylase provides additional molecular insights into enzyme catalysis and deficiency. *Biochim Biophys Acta.* 2016;1864(6):676-682.

9. Komori H, Nitta Y, Ueno H, Higuchi Y. Structural study reveals that Ser-354 determines substrate specificity on human histidine decarboxylase. *J Biol Chem.* 2012;287(34):29175-29183.

10. Fenalti G, Law RH, Buckle AM, et al. GABA production by glutamic acid decarboxylase is regulated by a dynamic catalytic loop. *Nat Struct Mol Biol.* 2007;14(4):280-286.

11. Montioli R, Bisello G, Dindo M, Rossignoli G, Voltattorni CB, Bertoldi M. New variants of AADC deficiency expand the knowledge of enzymatic phenotypes. *Arch Biochem Biophys.* 2020;682:108263.

12. Karamitros CS, Murray K, Winemiller B, et al. Leveraging intrinsic flexibility to engineer enhanced enzyme catalytic activity. *Proc Natl Acad Sci U S A.* 2022;119(23):e2118979119.

13. Bertoldi M, Frigeri P, Paci M, Voltattorni CB. Reaction specificity of native and nicked 3,4-dihydroxyphenylalanine decarboxylase. *J Biol Chem.* 1999;274(9):5514-5521.

14. Tancini B, Dominici P, Simmaco M, Schininà ME, Barra D, Voltattorni CB. Limited tryptic proteolysis of pig kidney 3,4-dihydroxyphenylalanine decarboxylase. *Arch Biochem Biophys.* 1988;260(2):569-576.

15. Bertoldi M, Borri Voltattorni C. Reaction and substrate specificity of recombinant pig kidney Dopa decarboxylase under aerobic and anaerobic conditions. *Biochim Biophys Acta.* 2003;1647(1-2):42-47.

16. Kass I, Hoke DE, Costa MG, et al. Cofactor-dependent conformational heterogeneity of GAD65 and its role in autoimmunity and neurotransmitter homeostasis. *Proc Natl Acad Sci U S A.* 2014;111(25):E2524-2529.

17. Bisello G, Kusmierska K, Verbeek MM, et al. The novel P330L pathogenic variant of aromatic amino acid decarboxylase maps on the catalytic flexible loop underlying its crucial role. *Cell Mol Life Sci.* 2022;79(6):305.

18. Montioli R, Dindo M, Giorgetti A, Piccoli S, Cellini B, Voltattorni CB. A comprehensive picture of the mutations associated with aromatic amino acid decarboxylase deficiency: from molecular mechanisms to therapy implications. *Hum Mol Genet.* 2014;23(20):5429-5440.

19. Longo C, Montioli R, Bisello G, et al. Compound heterozygosis in AADC deficiency: A complex phenotype dissected through comparison among heterodimeric and homodimeric AADC proteins. *Mol Genet Metab.* 2021.

20. Montioli R, Battini R, Paiardini A, et al. A novel compound heterozygous genotype associated with aromatic amino acid decarboxylase deficiency: Clinical aspects and biochemical studies. *Mol Genet Metab.* 2019;127(2):132-137.

21. Rossignoli G, Krämer K, Lugarà E, et al. Aromatic l-amino acid decarboxylase deficiency: a patient-derived neuronal model for precision therapies. *Brain.* 2021.

22. Montioli R, Cellini B, Borri Voltattorni C. Molecular insights into the pathogenicity of variants associated with the aromatic amino acid decarboxylase deficiency. *J Inherit Metab Dis.* 2011;34(6):1213-1224.

23. Montioli R, Janson G, Paiardini A, Bertoldi M, Borri Voltattorni C. Heterozygosis in aromatic amino acid decarboxylase deficiency: Evidence for a positive interallelic complementation between R347Q and R358H mutations. *IUBMB Life.* 2018;70(3):215-223.
